# Explainable Deep Multilevel Attention Learning for Predicting Protein Carbonylation Sites

**DOI:** 10.1002/advs.202500581

**Published:** 2025-03-27

**Authors:** Jian Zhang, Jingjing Qian, Pei Wang, Xuan Liu, Fuhao Zhang, Haiting Chai, Quan Zou

**Affiliations:** ^1^ School of Computer and Information Technology Xinyang Normal University Xinyang 464000 China; ^2^ Yangtze Delta Region Institute (Quzhou) University of Electronic Science and Technology of China Quzhou 324003 China; ^3^ Department of Anesthesia Critical Care and Pain Medicine Massachusetts General Hospital Harvard Medical School Boston MA 02114 USA; ^4^ College of Information Engineering Northwest A&F University Yangling Shaanxi 712100 China; ^5^ Nuffield Department of Medicine University of Oxford Oxford OX1 3SY UK

**Keywords:** attention mechanism, cross‐prediction, ligand interaction sites, protein carbonylation sites

## Abstract

Protein carbonylation refers to the covalent modification of proteins through the attachment of carbonyl groups, which arise from oxidative stress. This modification is biologically significant, as it can elicit modifications in protein functionality, signaling cascades, and cellular homeostasis. Accurate prediction of carbonylation sites offers valuable insights into the mechanisms underlying protein carbonylation and the pathogenesis of related diseases. Notably, carbonylation sites and ligand interaction sites, both functional sites, exhibit numerous similarities. The survey reveals that current computation‐based approaches tend to make excessive cross‐predictions for ligand interaction sites. To tackle this unresolved challenge, selective carbonylation sites (SCANS) is introduced, a novel deep learning‐based framework. SCANS employs a multilevel attention strategy to capture both local (segment‐level) and global (protein‐level) features, utilizes a tailored loss function to penalize cross‐predictions (residue‐level), and applies transfer learning to augment the specificity of the overall network by leveraging knowledge from pretrained model. These innovative designs have been shown to successfully boost predictive performance and statistically outperforms current methods. Particularly, results on benchmark testing dataset demonstrate that SCANS consistently achieves low false positive rates, including low rates of cross‐predictions. Furthermore, motif analyses and interpretations are conducted to provide novel insights into the protein carbonylation sites from various perspectives.

## Graphical abstract

Selective carbonylation sites (SCANS) are conceptualized, designed, evaluated, and released. SCANS captures segment‐level, protein‐level, and residue embeddings features. It utilizes elaborate loss function to penalize cross‐predictions at the residue level. SCANS is trained and optimized on comprehensive datasets that include protein carbonylation sites and various ligand interaction data. Furthermore, it applies transfer learning to enhance the specificity of the predictor.

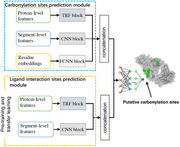

## 1 Introduction

Protein carbonylation is a common post‐translational modification. It occurs when reactive oxygen or nitrogen species interact with the side chains of certain amino acids, particularly those of lysine, arginine, proline, and threonine.^[^ ^1^ ^]^ The introduction of carbonyl groups, such as aldehydes and ketones, significantly affects protein structure, function, and stability.^[^ ^2^ ^]^ This modification alters the local conformation of proteins, making proteins become more susceptible to degradation.^[^ ^2^ ^]^ Furthermore, protein carbonylation influences the enzymatic activity and affects how proteins interact with other biomolecules, and influences signaling pathways. Carbonylated proteins accumulate with age, contributing to the aging process and related pathologies.^[^ ^3^ ^]^ They can lead to cellular dysfunction, which often occurs in various diseases. Recent studies have linked increased carbonylation to neurodegenerative such as Alzheimer's,^[^ ^4^ ^]^ Parkinson's^[^ ^5^ ^]^ and Huntington's^[^ ^6^ ^]^ disease, as well as cardiovascular diseases, including Atherosclerosis and heart failure,^[^ ^7^ ^]^ diabetes mellitus^[^ ^1^ ^]^ and etc. Particularly, since protein carbonylation can disrupt signaling pathways in cancer cells, affecting growth and survival, carbonylated proteins are usually seen to accumulate in tumor tissues.^[^ ^8^ ^]^

Understanding of protein carbonylation mechanism begins with the knowledge of carbonylation sites. Protein carbonylation occurs on a broader range of amino acids, including lysine (K), arginine (R), proline (P), threonine (T), histidine (H), cysteine (C), tryptophan (W).^[^ ^9^ ^]^ Accurately predicting protein carbonylation sites provides insights into how oxidative modifications influence protein structure and function, and helps elucidate the role of oxidative stress in related diseases. Moreover, it offers benefits for the development of drugs that specifically modulate these modifications. These modifications represent critical markers of cellular health and are valuable in research and therapeutic contexts. Biochemical techniques used to investigate protein carbonylation include dinitrophenylhydrazine (DNPH) assay, western blotting, mass spectrometry, enzyme‐linked immunosorbent assay, and fluorescent labeling.^[^ ^10^ ^]^ Although these biochemical experimental methods are effective, they come with limitations such as high cost, technical complexity, and an inability to provide real‐time assessments. Additionally, they also cannot catch up with the exponential growth of data generated by high‐throughput sequencing methods. Consequently, there is a growing interest in developing computational approaches to address these challenges. The breaking out of the artificial intelligence offers a promising avenue for inferring protein carbonylation sites directly from protein sequences using machine learning algorithms and data‐driven models.

We explore recent literature and identify a number of computation‐based predictors of protein carbonylation sites, including CarSitePred,^[^ ^11^ ^]^ Carsite_AGan,^[^ ^12^ ^]^ predML‐Site,^[^ ^13^ ^]^ CarSite‑II,^[^ ^14^ ^]^ iCarPS,^[^ ^15^ ^]^ PreCar_Deep,^[^ ^16^ ^]^ CarSPred2.0,^[^ ^17^ ^]^ CarSite,^[^ ^18^ ^]^ MDD‐carb,^[^ ^19^ ^]^ predCar‐site,^[^ ^20^ ^]^ Weng et al.,^[^ ^21^ ^]^ CarSPred.Y,^[^ ^22^ ^]^ iCar‐PseCp,^[^ ^23^ ^]^ and CarSPred.^[^ ^24^ ^]^ **Table** **1** summarizes the publication year, dataset, architecture, outputs, and performance measurement of these 14 methods. Among them, 6 (or 43%) of them were published in the past three years. Most have been made available to the research community as free web servers or software code. All these methods employ a window scheme, based on the premise that the characteristics of the adjacent neighboring residues provide informative clues for the prediction of the residue in the center of the window. The window length varies from a minimum of 7 to a maximum of 27 residues. Physicochemical properties and evolutionary profile are the two most used features to encode the carbonylation sites. Early studies relied on traditional machine learning algorithms, such as support vector machine (SVM) or random forest (RF), while more recent researchers have shifted toward deep learning frameworks. Most methods output both binary values and propensity scores, and thus utilize two corresponding evaluation metrics.

**Table 1:** Summary of computation‐based predictors of protein carbonylation sites.

Method^a)^	Year	Dataset includes other functional sites	Predictor^b)^	Architecture					Assess cross‐predictions	Outputs and performance measurement	
				Window	Physicochemical properties	Evolutionary profile	Protein language model	Predictive model^c)^		Binary values^d)^	Propensity scores^e)^
This work		√	SC	√	√	√	√	CNN, TRF	√	SN, SP, ACC, F1, MCC CPRratio, OPRratio, AULCratio	AUROC, AUPRC, AUCPC, AUOPC
CarSitePred^[^ ^11^ ^]^	2024	×	SC	√	√	√	×	CNN, LSTM	×	SN, SP, ACC, F1, MCC	AUROC, AUPRC
Carsite_AGan^[^ ^12^ ^]^	2023	×	SC	√	×	√	×	SVM	×	SN, SP	AUROC
predML‐Site^[^ ^13^ ^]^	2022	×	WS	√	√	√	×	SVM	×	SN, ACC	AUROC
CarSite‑II^[^ ^14^ ^]^	2021	×	WS	√	×	√	×	RF, SVM	×	SN, SP, ACC, MCC	AUROC
iCarPS^[^ ^15^ ^]^	2021	×	WS	√	√	√	×	RF	×	SN, SP, ACC, MCC	AUROC
PreCar_Deep^[^ ^16^ ^]^	2021	×	SC	√	√	×	×	CNN, LSTM	×	SN, SP, ACC	AUROC
CarSPred‐2.0^[^ ^17^ ^]^	2018	×	SC	√	√	×	×	SVM	×	SN, SP, ACC, MCC	AUROC
CarSite^[^ ^18^ ^]^	2017	×	N/A	√	√	×	×	SVM	×	SN, SP, ACC, MCC	×
MDD‐carb^[^ ^19^ ^]^	2017	×	WS	√	×	√	×	SVM	×	SN, SP, ACC, MCC	AUROC
predCar‐site^[^ ^20^ ^]^	2017	×	WS	√	×	×	×	SVM	×	SN, SP, PRE, ACC, MCC	AUROC
Weng et al.^[^ ^21^ ^]^	2017	×	N/A	√	√	√	×	SVM, DT, RF	×	SN, SP, ACC, MCC	×
CarSPred.Y^[^ ^22^ ^]^	2016	×	SC	√	√	×	×	SVM	×	SN, MCC	×
iCar‐PseCp^[^ ^23^ ^]^	2016	×	WS	√	×	×	×	RF	×	SN, SP, ACC, MCC	AUROC
CarSPred^[^ ^24^ ^]^	2014	×	SC	√	×	√	×	SVM	×	SN, SP, ACC, MCC	×

^a)^ The name of each method is either provided in the publication or the last name of its first author.

^b)^ SC: source code; WS: web server.

^c)^ CNN: convolutional neural networks; TRF: transformer; LSTM: long short‐term memory; SVM: support vector machine; RF: random forest; DT: decision tree.

^d)^ SN: sensitivity; SP: specificity; ACC: accuracy; MCC: Matthew's correlation coefficient; PRE: precision; F1: F1‐score.

^e)^ AUROC: area under the ROC curve; AUPRC: area under the precision recall curve; AUCPC: area under the cross‐prediction curve; AUPRC: area under the overprediction curve.

As far as we know, Table 1 reveals three drawbacks: *first*, the existing methods utilize a window scheme that incorporates feature such as physicochemical properties and evolutionary profile to build the feature space. None of them considers the integration of protein language model, which is trained on all protein sequences rather than small individual protein families, enhancing capture complex dependencies between amino acids; *second*, amino acids serve various functions, including undergoing post‐translational modifications and interacting with other small ligands. Unfortunately, these predictors were neither optimized nor assessed on datasets that include other functional sites. Recent studies have demonstrated that neglecting the influence of other interactions, the predictor shall be easily strapped into the cross‐predictions.^[^ ^25^ ^]^ The differences among functional sites are much less pronounced than those between functional and nonfunctional sites;^[^ ^25c^ ^]^ *third*, to construct an accurate and robust predictor, it is essential to assess the model using both binary values and propensity scores. Specifically, the outputs of the predictor should be evaluated for overpredictions, which predict nonfunctional residues as carbonylation sites, and cross‐predictions, which mispredict other functional sites as carbonylation sites.

Motivated by these drawbacks, our work focuses on this challenge to propose a novel predictor named selective carbonylation sites (SCANS) for accurately identifying protein carbonylation sites. Importantly, our SCANS incorporates several novel features: 1) We develop a new comprehensive benchmark dataset with experimentally verified protein carbonylation sites, ligand interaction sites, and nonfunctional residues. 2) We introduce a robust pretrained protein language model ESM2, designed to transfer semantic knowledge from large‐scale protein datasets to segments, facilitating the learning of high‐dimensional, long‐term features within these biological sequences. 3) We equip SCANS with multilevel attention mechanisms to capture segment‐level physicochemical properties and protein‐level evolutionary profile features. 4) Since carbonylation sites are easily mixed with ligand interaction sites, we implement transfer learning and a customized loss function to penalize cross‐predictions at the residue‐level. 5) To elucidate the predicting mechanisms of SCANS, we employ ablation analysis to address the limitations of “black‐box” approaches in deep neural network models, thereby enhancing the good interpretability of SCANS framework. 6) We test and compare the predictions on experimentally verified human carbonylated proteins. The benchmark datasets and source code of the SCANS method are available for academic use at https://github.com/jianzhang‐xynu/SCANS.

## 2 Experimental Section

### 2.1 Benchmark Datasets

In the present study, we leverage both carbonylation sites and ligand interaction sites to ensure comprehensive coverage in compiling the benchmark datasets. Protein carbonylation can occur at various amino acids. Consistent with previous research,^[^ ^11^, ^12^, ^13^, ^14^, ^15^ ^]^ we primarily predict four most prevalent types of carbonylation sites: lysine (K), arginine (R), proline (P), and threonine (T). The carbonylation annotations are sourced from CarbonylDB,^[^ ^9^ ^]^ which stores 1495 experimentally validated carbonylation proteins and 3781 carbonylation sites. We collect 1294, 457, 581, and 496 for K, P, R, and T carbonylation sites, respectively. Acknowledging that these four types of residues may also serve other functions, we incorporate residue‐level ligand interaction annotations. We gather ligand interaction information and PDB chains from the BioLiP2^[^ ^26^ ^]^ database. The PDB chains, along with the annotations, are mapped to the corresponding full UniProt sequences using SIFTS.^[^ ^27^ ^]^ This methodology has been shown to enhance the quality and completeness of ligand interaction data.^[^ ^28^ ^]^ As a result, we compile a dataset with 29992 ligand interaction proteins.

To compare with state‐of‐the‐art predictors, we select recent public web servers or source codes based on the following criteria: i) the predictors are publicly available as of November 2024; ii) they must successfully analyze protein sequences of average length (300 residues) within 20 min; and iii) their outputs should be evaluable using both binary values and propensity scores. We ultimately select four predictors: CarSitePred,^[^ ^11^ ^]^ iCarPS,^[^ ^15^ ^]^ CarSPred‐2.0,^[^ ^17^ ^]^ and CarSPred.^[^ ^24^ ^]^ To mitigate the potential influence of similar proteins, we use Blastclust with a cutoff of 25% to select nonredundant sequences from our precompiled datasets, which encompass both carbonylation proteins and ligand interaction proteins, alongside the training datasets of the aforementioned methods. This way we ensure that the newly compiled test data is fair for each of the considered predictors.

**Figure 1:**
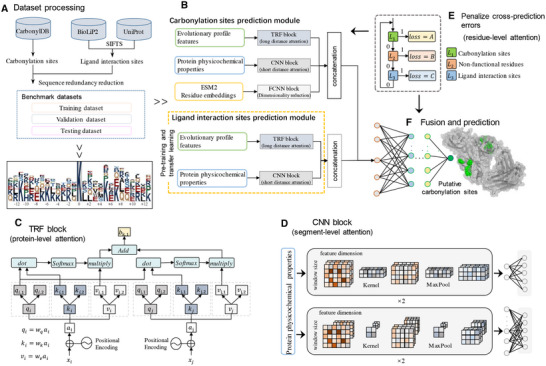
The overall workflow of SCANS. A) Dataset processing. We extract carbonylation sites from the CarbonylDB database. The structure chains and annotations from BioLiP2 are mapped onto the corresponding full UniProt sequences using SIFTS. This process yields high‐quality data on ligand interaction sites. After removing the homologous sequences, the benchmark dataset is divided into training, validation, and testing datasets. We conduct an analysis of amino acid position composition and carbonylation‐related motifs. B) SCANS utilizes a carbonylation sites prediction module and pretraining ligand interaction sites prediction module to identify functional sites. Both modules extract empirical features, including protein‐level evolutionary profile features and segment‐level physicochemical properties. The carbonylation sites prediction module also incorporates ESM2 to generate biological contextual embeddings. C) The CNN block employs short‐distance attention to learn segment‐level features. D) The TRF block utilizes long‐distance attention to learn protein‐level features. E) A customized loss function is designed to penalize cross‐prediction errors at the residue‐level. F) The outputs from the carbonylation sites prediction module and the ligand interaction sites prediction module are combined to further optimize the prediction of carbonylation sites.

We randomly pick 25% of the K, P, R, and T carbonylation sites. Accordingly, we use 374 or 25% proteins from a randomly selected nonredundant 1495 ligand interaction proteins to obtain the K, P, R, and T ligand interaction sites, as well as nonfunctional residues. This results in high quality benchmark testing datasets that include carbonylation sites, ligand interaction sites, and nonfunctional residues. The training and optimizing strategies involve splitting the rest data into benchmark training (comprising 80% of carbonylation sites and ligand interaction proteins) and validation (20% carbonylation sites and ligand interaction proteins) datasets. We follow procedures from related studies^[^ ^11^, ^15^ ^]^ by adopting the sliding window strategy to extract fixed‐length segments of 27 residues from the protein sequences, centering on the target sites. The dataset processing process is illustrated in **Figure** **1**A. **Table** **2** summarizes the datasets used in this study. The data is shared in the https://github.com/jianzhang‐xynu/SCANS.

**Table 2:** Summary of the datasets.

Carbonylation type	Type of residues	Training dataset	Validation dataset	Testing dataset	Carbonylation type	Type of residues	Training dataset	Validation dataset	Testing dataset
Lysine (K)	Carbonylation sites	654 (2.09%)	164 (2.09%)	323 (2.12%)	Proline (P)	Carbonylation sites	242 (2.09%)	61 (2.10%)	114 (2.04%)
	Ligand interaction sites	1603 (5.11%)	401 (5.12%)	923 (6.04%)		Ligand interaction sites	318 (2.74%)	80 (2.76%)	223 (3.98%)
	Nonfunctional	29094 (92.80%)	7274 (92.79%)	14025 (91.84%)		Nonfunctional	11043 (95.17%)	2761 (95.14%)	5259 (93.98%)
	ALL	31351	7839	15271		ALL	11603	2902	5596
Arginine (R)	Carbonylation sites	293 (2.07%)	74 (2.09%)	145 (2.09%)	Threonine (T)	Carbonylation sites	259 (2.05%)	65 (2.06%)	124 (2.07%)
	Ligand interaction sites	1131 (8.00%)	283 (8.00%)	522 (7.53%)		Ligand interaction sites	618 (4.89%)	154 (4.87%)	348 (5.80%)
	Nonfunctional	12722 (89.93%)	3181 (89.91%)	6266 (90.38%)		Nonfunctional	11769 (93.07%)	2942 (93.07%)	5525 (92.13%)
	ALL	14146	3538	6933		ALL	12646	3161	5997

Furthermore, to reach a consistent comparison with other methods, we also include a widely used dataset of 250 carbonylated proteins from Jia et al.^[^ ^23^ ^]^ This dataset contains 230 human carbonylated proteins and 20 Luminescent Bacillus and *E. coli* carbonylated proteins. In detail, it contains 300 K carbonylation sites and 1949 K noncarbonylation residues, 126 P carbonylation sites and 792 P noncarbonylation residues, 136 R carbonylation sites and 847 R noncarbonylation residues, and 121 T carbonylation sites and 732 T noncarbonylation residues, respectively.

### 2.2 Assessment of Predictive Performance

The proposed predictor generates two outputs: real‐valued propensities and 2‐bit binary scores. The former quantifies the likelihood that a given residue is a carbonylation site, while the latter uses a preset threshold to determine whether a given residue qualifies as a carbonylation site.

For the binary predictions, this study includes commonly used metrics: sensitivity/recall (SN), specificity (SP), accuracy (ACC), Matthews correlation coefficient (MCC), F1‐score (F1) are included in this study. They are defined as Equation ((1), (2), (3), (4), (5)). Specifically, TP, TN, FP, and FN indicate the number of true positives (correctly identified carbonylation sites), true negatives (correctly identified noncarbonylation sites), false positives (noncarbonylation sites incorrectly classified as carbonylation sites), and false negatives (carbonylation sites incorrectly classified noncarbonylation sites), respectively. Consistent with recently published related studies,^[^ ^25^, ^29^ ^]^ we further categorize the false positives into two groups and introduce the overprediction rate (OPR) and cross‐prediction rate (CPR) to differentiate two types of prediction errors. OPR stands for the fraction of nonfunctional residues predicted as carbonylation sites among all putative carbonylation sites; and the CPR indicates the fraction of ligand interaction sites predicted as carbonylation sites. Since the CPR and OPR values could be challenging to interpret (e.g., lower values indicate superior predictive performance), we calculate CPRratio and OPRratio. These ratios represent the CPR and OPR of a random predictor (or SN) divided by the corresponding CPR and OPR of a specified predictor, respectively. In this context, a ratio of 1 signifies that SN equals CPR or OPR, suggesting that this aspect of the prediction is equivalent to a random outcome. A ratio greater than 1 quantifies the degree of improvement over a random predictor (e.g., a ratio of 2 indicates performance that is twice better), while a ratio less than 1 denotes that the prediction performs worse than a random result.

(1) $$\mathrm{SN}\;=\frac{TP}{TP+FN}\;$$

(2) $$\mathrm{SP}\;=\frac{TN}{TN+FP}\;$$

(3) $$\mathrm{ACC}\;=\frac{TP+TN}{TP+FN+TN+FP}\;$$

(4) $$F1=\frac{2TP}{2TP+FP+FN}\;$$

(5) $$\begin{array}{c} \mathrm{MCC}\;=\frac{TP\times TN-FN\times FP}{\sqrt{\left(TP+FN\right)\times \left(TP+FP\right)\times \left(TN+FP\right)\times \left(TN+FN\right)}} \end{array}$$

(6) $$\mathrm{CPRratio}\;=\frac{SN}{CPR}\;$$

(7) $$\mathrm{OPRratio}\;=\frac{SN}{OPR}\;$$

For the numerical predictions, we estimate the predictive performance using receiver operating characteristic curve (ROC curve), precision recall curve (PR curve), cross‐prediction curve (CP curve), and overprediction curve (OP curve). We further quantify the effectiveness of different methods by calculating the area under the ROC curve (AUROC), area under the PR curve (AUPRC), area under the CP curve (AUCPC), and area under the OP curve (AUOPC). For the ROC curve, the AULC (Area Under the Low false positive rate ROC Curve) assesses the AUROC at a specified false positive rate (FPR). AULCratio represents the ratio of AULC compared with a random result. That is, AULCratio = 1 indicates a random result. A higher AULCratio reflects a superior model.

It is essential to analyze the significance of statistical differences between the proposed method and the current predictors. To achieve this, we randomly select 50% of the carbonylation sites, ligand interaction sites, and nonfunctional residues from the testing dataset, repeating this procedure 10 times. Initially, the Anderson‐Darling test is used to determine whether the predicted measurements are normally distributed. For data with a significance level of below 0.05 from the Anderson–Darling test, we employ the Wilcoxon rank‐sum test; otherwise, we use the Student's *t*‐test. The threshold for statistical significance is set at a *p*‐value of less than 0.001.

### 2.3 Selective of Enriched Carbonylation‐Related Motifs

Protein motifs are short conserved segments of amino acids that frequently occur within the same protein family.^[^ ^30^ ^]^ The conservation of motifs determines their stability during the evolutionary process, and involves biological activities. This characteristic allows motifs to serve as specific structural or function features in proteins.^[^ ^30^, ^31^ ^]^ In this work, we introduce information theory to compute and select carbonylation‐related motifs.^[^ ^19^ ^]^ First, we define *D_cs_* and *D_ncs_* as the dataset comprising carbonylation segments and randomly selected noncarbonylation segments from the training dataset, respectively. To mitigate data bias, the random selection is repeated 10 times. Each of the 10 *D_ncs_* is of the same size as *D_cs_*. The definitions of *D_cs_* and *D_ncs_* are as follows

(8) $$\left\{\begin{array}{ccc} D_{cs} & = & \left\{S_{cs}|S_{cs}^{i}\in \left\{CSs\right\}\right\}\\ D_{ncp}^{\tau } & = & \left\{S_{ncs}|S_{ncs}^{i}\in \left\{nCSs\right\}\right\}\\ i & \in & \left\{1,2,3,\text{…},m\right\}\\ \tau & \in & \left\{1,2,3,\text{…},10\right\} \end{array}\right.$$

where *S_cs_* and *S_ncs_* indicates the carbonylation segments and noncarbonylation segments, *i* denotes the *i*th segments, *m* represents the number of segments in the dataset, and τ stands for the τ‐th repeats. Next, we compute the information entropy of *D_cp_* and $D_{ncp}^{\tau }$, respectively

(9) $$\left\{\begin{array}{c} H\;\left(D_{cp}\right)=-\sum_{i}P\left(S_{cp}^{i}\right)\cdot logP\left(S_{cp}^{i}\right)\;\\ H\;\left(D_{ncp}^{\tau }\right)=-\sum_{i}P\left(S_{ncp}^{j}\right)\cdot logP\left(S_{ncp}^{j}\right) \end{array}\right.$$

We randomly generate sequence patterns *p_t_* with *l* residues. The length of *p_t_* is between 4 and *L*. In this study, *L* is empirically set as 10.

(10) $$p_{t}=\left\{AA|AA_{i=4}^{l}\in \left\{A,\;C,\text{…},\;Y\right\},4\leq l\leq L\right\}$$

Since the vast majority of these generated patterns do not appear in either the *D_cp_* or $D_{ncp}^{\tau }$ datasets. To avoid unnecessary computations, we preset a threshold of *T* = 5% to filter the generated patterns. We ensure that the occurrence frequencies of *p_t_* in *D_cp_* are greater than *T*, while the average frequencies in *D_ncp_* remain below *T*. Subsequently, we use the selected *p_t_* to reclassify the data and update the information entropy

(11) $$f\left(p_{t},D_{cp}\right)>T>f\left(p_{t},D_{ncp}^{\tau }\right)$$

(12) $$\begin{array}{c} H^{\prime }\;\left(D,p_{t}\right)=f\left(p_{t},D\right)\cdot H\left(p_{i}|p_{t}\right)+\left(1-f\left(p_{t},D\right)\right)\cdot H\left(p_{i}|\bar{p_{t}}\right) \end{array}$$

The information gain of *p_t_* is determined by comparing the original information entropy with the updated entropy. We compute the information gain for both *D_cp_* and *D_ncp_*. The term *IGR*(*p_t_*) represents the relatively information gain ratio introduced by *p_t_*

(13) $$IG\;\left(D,p_{t}\right)=H\left(D\right)-H^{\prime }\left(D,p_{t}\right)$$

(14) $$IGR\;\left(p_{t}\right)=\frac{IG\left(D_{cp},p_{t}\right)}{IG\left(D_{ncp},p_{t}\right)}\;$$

A higher *IGR*(*p_t_*) score indicates greater discriminatory power in distinguishing between carbonylation segments and noncarbonylation segments. Finally, we sort *IGR*(*p_t_*) and select top *q* motifs.

(15) $$motifs=\left\{p_{1},p_{2},\text{…}p_{q}\right\}\;$$

### 2.4 Architecture of the SCANS Predictor

#### 2.4.1 Empirical Features and Residue Embeddings

Effective identification of carbonylation sites hinges on the extraction of relevant features from protein sequences. These features enable the computational models to discern special patterns associated with carbonylation sites. Motivated by the survey in Table 1, this study incorporates evolutionary profile features and physicochemical properties. Specifically, we utilize MMseqs2^[^ ^32^ ^]^ with default parameters to search the UniProt30 dataset, obtaining the position specific scoring matrix (PSSM) for the target protein. Studies in^[^ ^21^, ^33^ ^]^ demonstrate that the PSSM can be used to infer the evolutionary level, highlighting conservation and variability, as well as the function and structural characteristics of the sequence. The PSSM is represented as an *L* × 20 scoring matrix, where the score at each position stands for the ratio of the observed frequency of that amino acid at the position to the background frequency across all positions. We extract the PSSM with a window length of 27 centered on the target site/residue (same as fixed‐length of the protein segments) as the evolutionary profile features. The physicochemical properties of residues collectively determine protein structure, stability, function, and ability to interact with other molecules.^[^ ^34^ ^]^ For instance, hydrophilic residues tend to locate on protein surface, maintaining their water solubility. Conversely, hydrophobic residues prefer to cluster inside proteins, forming a hydrophobic core that stabilizes its structure. We empirically select ten physicochemical properties: hydrophilicity, hydrophobicity, tiny, acidity, positively charged, negatively charged, polarity, aromaticity, sulfur content, aliphatic, as shown in Table S1 (Supporting Information). However, empirical features are insufficient to encode protein carbonylation sites. Thus, we extend the model to consider the evolutionary scale by incorporating Evolutionary Scale Modeling (ESM),^[^ ^35^ ^]^ a self‐attention‐based protein language representation model within our proposed deep network framework. ESM computes the biological contextual information (i.e., residue embeddings) for protein sequences. ESM‐generated embeddings have proven effective in predicting protein structure and function.^[^ ^36^ ^]^ This study we adopt the smallest model (esm2_t6_8M_UR50D with 8 M parameters) to capitalize on the higher variance of its predictions and mitigate the risk of overfitting.

#### 2.4.2 Multilevel Attention Networks

The overall network architecture of the deep learning network of SCANS is illustrated in Figure 1B with two main modules that work together to predict functional sites: the Carbonylation Sites Prediction Module (CSPM) and the Ligand Interaction Sites Prediction Module (LISPM). The CSPM is structured with a transformer (TRF) block, a convolutional neural network (CNN) block, and a fully connected neural network (FCNN) block. For the protein‐level evolutionary profile features, we introduce the TRF block that is composed of three stacked TRFs. These TRFs enhance training efficiency and effectively utilize sequence order information. Each TRF is composed of a self‐attention unit, a feedforward layer, and a normalization layer

(16) $$\begin{array}{c} output=MultiHead\;\left(Q,K,V\right)=Concat\left(head_{1},\text{…},head_{h}\right)W^{O} \end{array}$$

(17) $$head_{1}=Attention\left(Q,K,V\right)$$

(18) $$Attention\;\left(Q,K,V\right)=softmax\left(\frac{QW^{T}}{\sqrt{d_{k}}}\right)\cdot V$$

(19) $$\left\{\begin{array}{c} Q=X\cdot W_{i}^{Q}\\ K=X\cdot W_{i}^{K}\\ V=X\cdot W_{i}^{V} \end{array}\right.$$

where *Q*, *K*, *V* denote Query, Key, and Value, respectively, which are projected through *i* distinct transformer encoder. The term $\sqrt{d_{k}}$ serves as the scaling factor to regulate the magnitude of the dot product operations. The sets $\left\{W_{i}^{Q},W_{i}^{K},W_{i}^{V}\right\}$ denote the learned weight matrix for the *i*th head, *X* represents the input features, and *W^O^* is the learned weight matrix for the final linear projection of multihead outputs. Figure 1C shows the multihead protein‐level attention mechanism. In addition to empirical features, the high‐dimension embeddings generated from ESM2^[^ ^35^ ^]^ are fed into the FCNN block, consisting of two layers, which reduces the dimension to 10 to eliminate irrelevant information. Finally, the feature space generated by the three blocks are merged together.

We utilize the CNN block to process segment‐level physicochemical properties, which characterize the microscopic biochemical environment near the interaction site. This block features a two‐layer CNN‐1D and CNN‐2D, enabling it to capture relations between neighboring residues in the input sequence segments, as shown in Figure 1D. The outputs are subsequently normalized and passed through a Rectified Linear Unit (ReLU) activation unit,^[^ ^37^ ^]^ followed by a max pooling layer that aggregates higher dimensional information, as detailed follows

(20) $$S_{pc}=\left[{S_{pc}}_{-w},\text{…},{S_{pc}}_{-1},{S_{pc}}_{0},{S_{pc}}_{1},\text{…},{S_{pc}}_{w}\right]$$

(21) $$F_{C}\left(S_{pc}\right)=ReLU\left(BN\left(Conv\left(S_{pc}\right)\right)\right)$$

(22) $$Conv\;\left(S_{pc}\right)=\left\{\begin{array}{c} \begin{array}{cc} \sum_{i=1}^{l}K_{c,i}\cdot {S_{pc}}_{n,i-p_{l}} & p_{l}<i<p_{l}+l \end{array}\\ \begin{array}{cc} 0\; & otherwise \end{array} \end{array}\right.\;$$

(23) $$BN\;\left(X\right)=\gamma \left(\frac{x-E\left(X\right)}{\sqrt{Var\left(X\right)+\varepsilon }}\right)+\beta$$

(24) $$ReLU\;\left(x\right)=\left\{\begin{array}{c} \begin{array}{cc} x\; & x>0 \end{array}\\ \begin{array}{cc} 0\; & otherwise \end{array} \end{array}\right.\;$$

where *S_pc_* represents the segment with centered K/P/R/T ligand interaction site and *w*‐length residues on both sides. *Conv*(*S_pc_*) denotes a CNN‐1D or CNN‐2D layer equipped with a learnable convolutional kernel *K* of *l* channels and a padding *p_l_*. *BN*(*X*) stands for a normalization layer that incorporates two learnable parameters, γ and β. The terms *E*(*X*) and *Var*(*X*) denote the mini‐batch mean and variance, respectively; the ε serves as a small constant added to ensure numerical stability. The ReLU activation function is utilized to facilitate efficient gradient propagation and to accelerate the convergence process of the model. For the CNN‐1D block, we employ a 2‐layer CNN‐1D architecture with the following parameters: kernel size = 2, stride = 1, MaxPool kernel size = 2, and dropout rate = 0.5. For the CNN‐2D block, we also implement a 2‐layer CNN‐2D architecture with these parameters: kernel size = (2, 2), stride = 1, MaxPool kernel size = (2, 2), and dropout rate = 0.5.

The LISPM employs similar CNN and TRF block to capture the local and global features of ligand interaction sites. We use transfer learning to train the LSPM, enabling it to learn both segment‐level and protein‐level features, thereby competing the pretraining phase. The feature spaces generated by the CSPM and LISPM are combined and fed into a four‐layer FCNN, with the objective of improving the accuracy of the carbonylation sites prediction by minimizing the cross‐predictions with the help of the ligand interaction sites predictions (Figure 1F). The final output is activated by the sigmoid function, providing the probability of putative carbonylation sites.

#### 2.4.3 Transfer Learning

We design SCANS to accurately predict protein carbonylation sites while eliminating the influence of ligand interaction sites. The training and validation datasets are used to train and optimize the model, and the testing dataset is employed to evaluate the accuracy and generalization performance of the model. We pretrain the ligand interaction module using the training set annotated with the ligand interaction sites (i.e., we set the carbonylation sites as negatives). This module is optimized using the binary cross‐entropy loss function, aiming to maximize the AUROC value on the validation dataset. To avoid potential overfitting, we ensure that the difference in AUROC values between the training set and the validation set differs by no more than 3%. The ligand interaction module is designed to refine the prediction of carbonylation sites by reducing the cross‐predictions (prediction of carbonylation sites for the putative ligand interaction sites). We achieve this by freezing the LISPM when we subsequently train SCANS (i.e., we “transfer” the pretrained networks into the SCANS model).

### 2.5 Loss Function

SCANS aims to correctly recognize carbonylation sites and reduce potential cross‐prediction errors. Since the traditional binary cross‐entropy loss function does not distinguish between different types of errors, we design a customized focal loss by introducing α and β coefficients to effectively balance cross‐prediction errors and overprediction errors (Figure 1E), as follows

(25) $$\begin{array}{c} Loss=-{\left(1-p\right)}^{r}\;l_{c}\log\left(p\right)-\alpha \cdot p^{r}l_{n}log\left(1-p\right)-\beta \cdot p^{r}l_{i}log\left(1-p\right) \end{array}$$

where *p* indicates the predicted carbonylation site propensities; *r* is used to diminish the relative loss for well‐classified samples;^[^ ^38^ ^]^ *l_c_*, *l_n_*, and *l_i_* stand for carbonylation site, nonfunctional residue and ligand interaction site labels, respectively. The coefficients α and β represent the different attentions allocated to cross‐prediction errors and overprediction errors in the loss computation. When coefficient α is assigned a higher value, it indicates that the model places greater emphasis on cross‐prediction accuracy, thereby exhibiting a stronger tendency to penalize cross‐prediction errors. Conversely, when coefficient β is assigned a higher value, it signifies that the model demonstrates a heightened focus on overprediction errors, prioritizing the mitigation of such prediction inaccuracies in its optimization process. We fine‐tune α and β to optimize a good predictive accuracy and minimize cross‐prediction errors on the training and validation dataset. This architecture is trained using PyTorch with the Adam optimizer, setting the learning rate and batch size to 0.001 and 256, respectively.

## 3 Results and Discussions

### 3.1 Analysis of Amino Acid Position Specificity and Carbonylation‐Related Motifs

**Figure 2:**
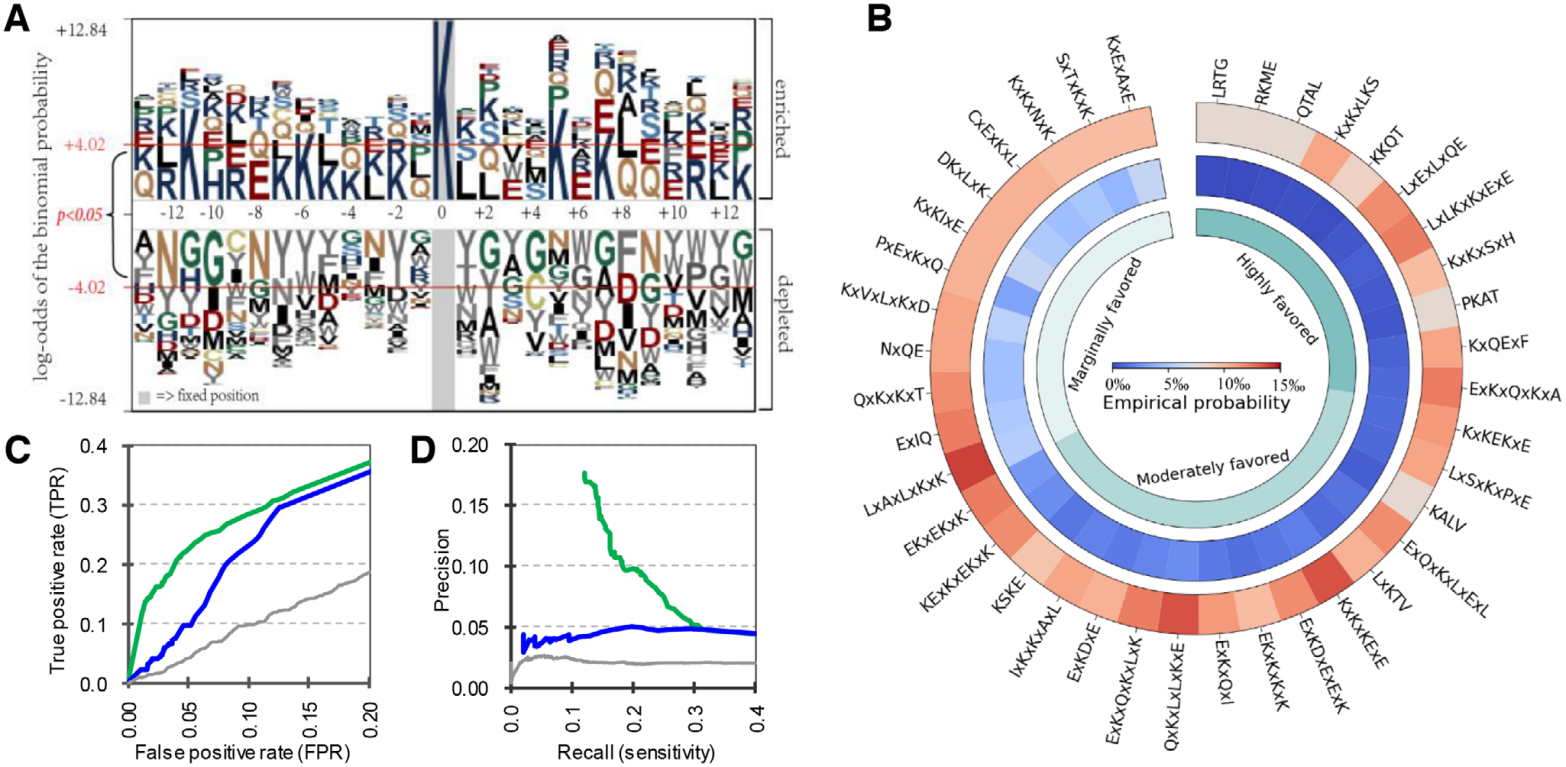
Amino acid position specificity and carbonylation‐related motifs. A) Sequence logo of lysine. B) The Circos heatmap of the empirical probabilities of selected motifs on lysine carbonylation segments versus noncarbonylation segments. C) The ROC curves (provided for the FPR < 0.2) of the motif‐based model. D) The PR curves (provided for the TPR < 0.2) of the motif‐based model.

We utilize pLogo^[^ ^39^ ^]^ to investigate and illustrate the position‐specific propensities in the amino acid distributions of carbonylation fragments compared to noncarbonylation fragments. In **Figure** **2**A, we observe a significant enrichment of lysine at positions −11, −7, −6, −5, +5, and +7, while glutamic acid is more frequently distributed at positions −8 and +3, consistent with findings reported by Kao et al.^[^ ^19^ ^]^ Both lysine and glutamic acid are categorized as charged and polar amino acids. Glutamine, another polar amino acid, shows slight enrichment at positions −1, +8, and +9, while leucine exhibits higher frequency at positions −3, +1, and +2. Additionally, we note a significant depletion of tryptophan, tyrosine, glycine, and asparagine in the carbonylation segments. Among these, tryptophan and tyrosine are classified as aromatic amino acids. The amino acid propensities in the carbonylation fragments prompt us to consider whether this enrichment contributes to the formation of motifs associated with carbonylation.

As detailed in Section 2.3, we calculate and present the Circos heatmap in Figure 2B. The motifs are ranked according to their IGR values, and we further categorized the selected motifs into three groups: highly favored (IGR > 10), moderately favored (2 < IGR ≤ 10), and marginally favored (1 ≤ IGR < 2). The top three motifs are “*LRTG*”, “*RKME*”, and “*QTAL*”, each with an IGR greater than 50. Furthermore, we observe that “*KxKxLKS*”, “*LxExLxQE*”, “*LxLKxKxExE*”, and “*ExKxQxKxA*” not only have a high probability of occurrence in protein carbonylation segments but also exhibit notably high IGR values. Subsequently, we employ these motifs to develop a straightforward predictive tool aimed at identifying carbonylation segments. We utilize cumulative IG values to quantify the likelihood of each motif's occurrence within the protein segments. **Table** **3** presents an evaluation of the motif‐based method's performance across four carbonylation testing datasets, yielding average AUROC values ranging from 0.58 to 0.61. The observed low AUROC values can be attributed to the fact that the majority of protein segments lack the selective motifs, thereby limiting the model's predictive capability for those segments. However, when these motifs are present in specific protein segments, the motif‐based approach proves effective. This phenomenon is further corroborated by Figure 2C,D, which illustrate the ROC curves (FPR ≤ 0.2) and PR curves (TPR ≤ 0.2) for the K carbonylation training dataset (green) and testing dataset (blue), respectively. Notably, when the FPR is set at 10%, the TPR on the testing dataset reaches 22%, which is 2.2 times the average level. The PR curve also illustrates that the predictive performance of the selected motifs significantly exceeds random level (grey). These experiments validate the efficacy of the computationally derived motifs and confirm the rationale behind incorporating evolutionary profile to encode protein carbonylation segments. The experimental results for P, R, and T carbonylation‐related motifs are presented in Figures S1–S4 (Supporting Information). The selected carbonylation‐related motifs can be found in Table S2 (Supporting Information) and at https://github.com/jianzhang‐xynu/SCANS.

**Table 3:** Predictive performance of SCANS and current methods on the benchmark testing datasets. We evaluate robustness across different datasets by conducting 10 tests on randomly selected 50% samples from the test dataset. The results are presented as averages ± standard deviations, along with *p*‐values comparing SCANS to each of the methods. The best results are highlighted in bold font.

Carbonylation type	Method	SN		AULCratio		MCCmax	F1max	AUROC	AUPRC
		SP = 95%	SP = 90%	SP = 95%	SP = 90%				
Lysine (K)	CarSPred	0.037 ± 0.007	0.094 ± 0.014	0.498 ± 0.100	0.833 ± 0.113	0.014 ± 0.006	0.044 ± 0.002	0.485 ± 0.014	0.020 ± 0.001
		*p* = 3.4E‐26	*p* = 2.0E‐24	*p* = 4.4E‐25	*p* = 2.2E‐27	*p* = 1.2E‐22	*p* = 1.8E‐22	*p* = 3.9E‐22	*p* = 8.7E‐22
	CarSPred‐2.0	0.068 ± 0.020	0.130 ± 0.019	1.258 ± 0.434	1.337 ± 0.271	0.026 ± 0.006	0.052 ± 0.006	0.528 ± 0.021	0.024 ± 0.002
		*p* = 7.0E‐23	*p* = 4.5E‐23	*p* = 3.0E‐23	*p* = 1.2E‐24	*p* = 2.7E‐22	*p* = 6.2E‐22	*p* = 8.2E‐19	*p* = 1.3E‐21
	iCarPS	0.052 ± 0.011	0.132 ± 0.017	0.825 ± 0.213	1.125 ± 0.174	0.069 ± 0.007	0.064 ± 0.002	0.627 ± 0.011	0.029 ± 0.001
		*p* = 5.0E‐25	*p* = 2.8E‐23	*p* = 1.4E‐24	*p* = 2.5E‐26	*p* = 2.9E‐21	*p* = 5.7E‐22	*p* = 7.3E‐19	*p* = 1.7E‐21
	CarSitePred	0.041 ± 0.013	0.074 ± 0.018	0.773 ± 0.257	0.768 ± 0.208	0.036 ± 0.008	0.051 ± 0.003	0.528 ± 0.014	0.022 ± 0.001
		*p* = 6.7E‐25	*p* = 5.4E‐24	*p* = 2.0E‐24	*p* = 4.4E‐26	*p* = 8.0E‐22	*p* = 3.1E‐22	*p* = 4.2E‐21	*p* = 1.1E‐21
	Motif‐based	0.099 ± 0.023	0.223 ± 0.017	1.952 ± 0.345	2.185 ± 0.259	0.071 ± 0.009	0.083 ± 0.006	0.582 ± 0.010	0.037 ± 0.220
		*p* = 9.0E‐21	*p* = 1.5E‐21	*p* = 3.4E‐20	*p* = 1.2E‐21	*p* = 4.5E‐19	*p* = 3.4E‐19	*p* = 6.0E‐20	*p* = 8.2E‐16
	SCANS	**0.594 ± 0.016**	**0.692 ± 0.019**	**17.612 ± 0.614**	**10.924 ± 0.249**	**0.389 ± 0.018**	**0.401 ± 0.018**	**0.823 ± 0.011**	**0.266 ± 0.014**
Proline (P)	CarSPred	0.060 ± 0.021	0.098 ± 0.031	0.937 ± 0.378	0.977 ± 0.303	0.030 ± 0.007	0.050 ± 0.005	0.511 ± 0.021	0.021 ± 0.002
		*p* = 5.6E‐19	*p* = 2.2E‐19	*p* = 1.7E‐16	*p* = 1.2E‐18	*p* = 8.9E‐18	*p* = 5.6E‐17	*p* = 1.2E‐19	*p* = 4.5E‐13
	CarSPred‐2.0	0.044 ± 0.015	0.105 ± 0.035	0.930 ± 0.282	0.928 ± 0.298	0.043 ± 0.013	0.051 ± 0.006	0.508 ± 0.021	0.022 ± 0.002
		*p* = 1.2E‐19	*p* = 7.1E‐19	*p* = 1.4E‐16	*p* = 1.1E‐18	*p* = 4.6E‐17	*p* = 6.3E‐17	*p* = 9.5E‐20	*p* = 4.7E‐13
	iCarPS	0.083 ± 0.020	0.128 ± 0.022	1.391 ± 0.343	1.355 ± 0.260	0.069 ± 0.012	0.069 ± 0.006	0.614 ± 0.020	0.028 ± 0.003
		*p* = 1.1E‐18	*p* = 5.7E‐20	*p* = 2.6E‐16	*p* = 1.8E‐18	*p* = 1.7E‐16	*p* = 1.7E‐16	*p* = 2.4E‐17	*p* = 7.4E‐13
	CarSitePred	0.037 ± 0.013	0.144 ± 0.034	0.93 ± 0.317	1.125 ± 0.274	0.048 ± 0.011	0.058 ± 0.008	0.556 ± 0.022	0.024 ± 0.002
		*p* = 7.3E‐20	*p* = 1.7E‐18	*p* = 1.5E‐16	*p* = 1.3E‐18	*p* = 4.2E‐17	*p* = 1.1E‐16	*p* = 2.5E‐18	*p* = 5.4E‐13
	Motif‐based	0.144 ± 0.043	0.241 ± 0.050	3.021 ± 0.863	2.826 ± 0.638	0.087 ± 0.023	0.098 ± 0.020	0.602 ± 0.026	0.032 ± 0.004
		*p* = 1.6E‐14	*p* = 4.8E‐15	*p* = 3.3E‐15	*p* = 1.6E‐15	*p* = 7.0E‐15	*p* = 2.9E‐15	*p* = 1.5E‐16	*p* = 3.2E‐13
	SCANS	**0.605 ± 0.038**	**0.721 ± 0.035**	**16.980 ± 1.725**	**10.903 ± 0.769**	**0.339 ± 0.031**	**0.371 ± 0.033**	**0.885 ± 0.018**	**0.275 ± 0.044**
Arginine (R)	CarSPred	0.125 ± 0.024	0.175 ± 0.031	2.891 ± 0.456	2.323 ± 0.389	0.067 ± 0.010	0.085 ± 0.010	0.563 ± 0.028	0.031 ± 0.005
		*p* = 1.7E‐18	*p* = 7.7E‐18	*p* = 3.2E‐19	*p* = 7.5E‐19	*p* = 1.1E‐19	*p* = 1.1E‐19	*p* = 1.0E‐16	*p* = 3.5E‐18
	CarSPred‐2.0	0.062 ± 0.015	0.107 ± 0.021	1.942 ± 0.508	1.277 ± 0.283	0.056 ± 0.015	0.067 ± 0.013	0.511 ± 0.019	0.025 ± 0.003
		*p* = 1.0E‐19	*p* = 2.1E‐19	*p* = 1.8E‐19	*p* = 9.0E‐20	*p* = 1.5E‐19	*p* = 8.7E‐20	*p* = 2.5E‐19	*p* = 2.8E‐18
	iCarPS	0.108 ± 0.035	0.197 ± 0.032	2.062 ± 0.721	2.091 ± 0.487	0.077 ± 0.013	0.078 ± 0.010	0.629 ± 0.029	0.034 ± 0.005
		*p* = 6.2E‐18	*p* = 1.6E‐17	*p* = 3.7E‐19	*p* = 1.1E‐18	*p* = 2.1E‐19	*p* = 8.4E‐20	*p* = 8.2E‐15	*p* = 3.8E‐18
	CarSitePred	0.028 ± 0.013	0.091 ± 0.016	0.412 ± 0.187	0.749 ± 0.138	0.020 ± 0.008	0.045 ± 0.004	0.489 ± 0.025	0.020 ± 0.001
		*p* = 3.8E‐20	*p* = 7.7E‐20	*p* = 3.8E‐20	*p* = 2.4E‐20	*p* = 2.1E‐20	*p* = 1.9E‐20	*p* = 8.7E‐19	*p* = 2.3E‐18
	Motif‐based	0.170 ± 0.030	0.268 ± 0.039	4.048 ± 0.651	3.371 ± 0.436	0.102 ± 0.015	0.114 ± 0.014	0.604 ± 0.018	0.046 ± 0.005
		*p* = 4.8E‐19	*p* = 4.8E‐17	*p* = 4.4E‐21	*p* = 4.8E‐20	*p* = 5.5E‐21	*p* = 2.5E‐22	*p* = 1.3E‐18	*p* = 2.4E‐20
	SCANS	**0.761 ± 0.048**	**0.782 ± 0.046**	**27.644 ± 1.856**	**14.627 ± 0.917**	**0.651 ± 0.041**	**0.650 ± 0.040**	**0.900 ± 0.023**	**0.626 ± 0.052**
Threonine (T)	CarSPred	0.073 ± 0.016	0.139 ± 0.029	1.990 ± 0.517	1.459 ± 0.293	0.092 ± 0.022	0.068 ± 0.014	0.515 ± 0.021	0.031 ± 0.004
		*p* = 6.1E‐20	*p* = 1.5E‐17	*p* = 5.5E‐20	*p* = 7.3E‐20	*p* = 2.9E‐20	*p* = 6.9E‐22	*p* = 1.3E‐17	*p* = 4.1E‐18
	CarSPred‐2.0	0.137 ± 0.034	0.179 ± 0.039	3.118 ± 0.962	2.282 ± 0.562	0.097 ± 0.031	0.090 ± 0.021	0.542 ± 0.025	0.035 ± 0.008
		*p* = 1.3E‐17	*p* = 2.4E‐16	*p* = 1.4E‐18	*p* = 3.5E‐18	*p* = 1.4E‐18	*p* = 2.1E‐20	*p* = 1.5E‐16	*p* = 8.2E‐18
	iCarPS	0.073 ± 0.014	0.177 ± 0.037	1.271 ± 0.317	1.599 ± 0.285	0.073 ± 0.012	0.072 ± 0.008	0.626 ± 0.024	0.030 ± 0.003
		*p* = 4.5E‐20	*p* = 1.5E‐16	*p* = 1.5E‐20	*p* = 8.5E‐20	*p* = 3.3E‐22	*p* = 1.1E‐22	*p* = 1.6E‐14	*p* = 3.9E‐18
	CarSitePred	0.050 ± 0.016	0.100 ± 0.025	0.812 ± 0.326	0.980 ± 0.262	0.020 ± 0.006	0.046 ± 0.005	0.466 ± 0.034	0.020 ± 0.002
		*p* = 3.4E‐20	*p* = 2.5E‐18	*p* = 1.1E‐20	*p* = 2.8E‐20	*p* = 8.3E‐24	*p* = 2.3E‐23	*p* = 6.9E‐17	*p* = 2.2E‐18
	Motif‐based	0.240 ± 0.052	0.297 ± 0.041	5.440 ± 1.167	4.802 ± 0.968	0.139 ± 0.025	0.148 ± 0.024	0.612 ± 0.022	0.058 ± 0.011
		*p* = 2.2E‐14	*p* = 1.4E‐15	*p* = 4.5E‐17	*p* = 3.0E‐14	*p* = 7.8E‐17	*p* = 4.4E‐17	*p* = 8.0E‐17	*p* = 5.1E‐15
	SCANS	**0.700 ± 0.041**	**0.747 ± 0.050**	**23.403 ± 1.406**	**13.156 ± 0.778**	**0.553 ± 0.022**	**0.561 ± 0.023**	**0.881 ± 0.027**	**0.381 ± 0.031**

### 3.2 Evaluation of Window Size Selection

The adjacent residues to the carbonylation sites have an influence on the protein carbonylation process. These influences can be quantified by adjusting the length of the window during model construction. We build feature spaces and fine‐tune models on the training and validation sets using window sizes of 5, 7, 9, 11, 13, 15, 17, 19, and 21. For each window size, we select the model aiming to maximize the AUROC value on the validation dataset, ensuring that the difference in AUROC values between the training set and the validation set remains within 3%.

Figure S5 (Supporting Information) illustrates the predictive performance of the model on four validation datasets with varying window sizes. As shown in Figure S5A (Supporting Information), for the carbonylation validation dataset centered on lysine K, the AUROC value increases from 0.84 to over 0.89, representing a 0.05 increase (or 6% improvement). Meanwhile, the AUCPC value decreases from ≈0.09 to 0.06, which is a reduction of 0.03 (or 33% decrease). This suggests that as the window size increases, more informative clues are incorporated into the predictive model. Consequently, the model is capable of identifying more carbonylation sites. However, a longer window size does not certainly correlate with better results. When the window size exceeds 13 in Figure S5A (Supporting Information), performance on the validation set faces a slight decline in AUROC (from 0.89 to 0.87) and a minor increase in AUCPC (from 0.06 to 0.07). Similar trends are also observed in the proline P (Figure S5B, Supporting Information), arginine R (Figure S5C, Supporting Information), and threonine T (Figure S5D, Supporting Information) carbonylation validation datasets. Based on these findings, we have selected window sizes of 13, 15, 17, and 17 as the optimal window sizes for the K, P, R, and T carbonylation models, respectively. These choices aim to balance the inclusion of relevant information with the avoidance of overfitting or diminishing returns in model performance.

### 3.3 Exploration of the Optimal Parameters of the Loss Function

In this study, we build a deep multilevel attention learning framework to train and optimize the predictive model. We begin by pretrain the ligand interaction sites prediction module using binary cross‐entropy loss function. Next we transfer and freeze the pretrained network when we subsequently train SCANS with a customized loss function as detailed in Section 2.5. Similar to Section 3.4 we select the model with highest AUROC values on the validation dataset and ensure that the differences in AUROC values between the training set and the validation set are less than 3%.

**Figure 3:**
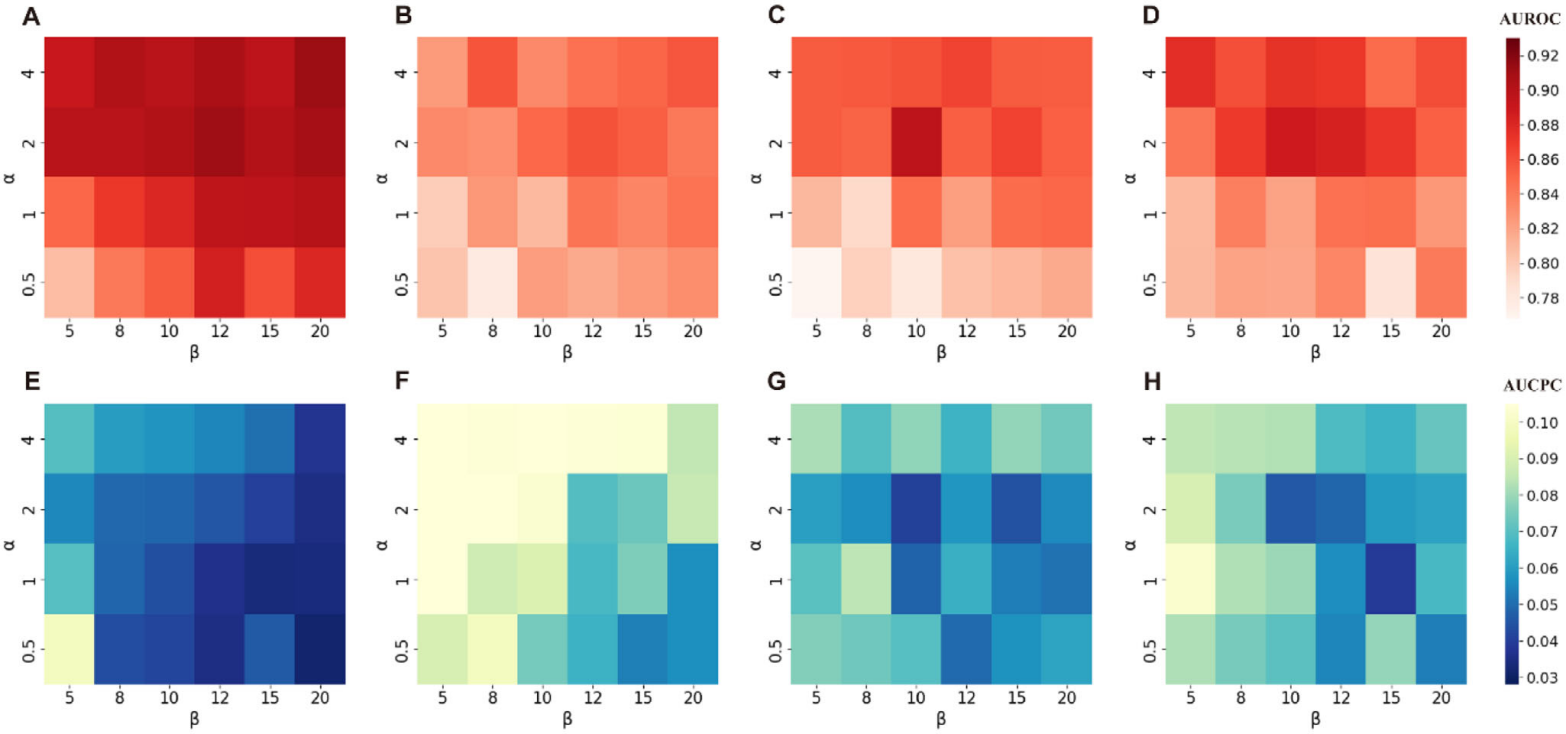
The heatmaps of the predictive performance for the four validation datasets using grid search to identify optimal loss function parameters.

**Figure** **3** provides a visual representation of how predictive performance varies with different combinations of α and β in the loss function. The *y*‐axis represents the α coefficient, and the *x*‐axis represents the β coefficients. A higher α indicates greater penalties for over‐prediction errors, such as misidentifying nonfunctional residues as carbonylation sites. Conversely, a higher β suggests stronger penalties for cross‐prediction errors, where ligand interaction sites are incorrectly recognized as carbonylation sites. Figure 3 panels A–D display the AUROC values, with darker colors indicating more accurate predictions. Correspondingly, panels E‐H present the AUCPC values, where darker colors mean lower cross‐prediction errors.

Obviously, as α increases, the model places greater emphasis on identifying the vast majority of negative samples (nonfunctional residues), resulting in a corresponding rise in AUROC values. However, this improvement does not guarantee that the model will effectively filter out ligand interaction sites. For example, when β is set to 8 in the carbonylation K model, the AUROC increases from 0.84 to 0.90, while the AUCPC synchronously increases from 0.04 to 0.06. Therefore, the training and optimizing process must consider the β parameter, which is designed to penalize the misclassification of ligand‐binding sites as carbonylation sites.

We select the optimal parameters based on two criteria: 1) achieving the highest AUROC value, and 2) ensuring that the differences between the corresponding AUCPC values and the lowest AUCPC value in the grid are less than 2%. Ultimately, the optimized loss function parameters for the four models are as follows: α = 2 and β = 12 for the lysine K carbonylation predictive model, α = 2 and β = 12 for the proline P carbonylation predictive model, α = 2 and β = 10 for the arginine R carbonylation predictive model, and α = 2 and β = 10 for the threonine T carbonylation predictive model.

### 3.4 Comparison with Existing Prediction Models on Benchmark Datasets

We first assess the validity of the proposed deep learning framework (excluding pretraining LISPM) using Jia's widely recognized dataset of 250 carbonylated proteins. Our method is compared against with PTMPred,^[^ ^40^ ^]^ CarSpred,^[^ ^24^ ^]^ iCar‐PseCp,^[^ ^23^ ^]^ CarSite,^[^ ^18^ ^]^ CarSite‐II,^[^ ^14^ ^]^ Carsite_AGan^[^ ^12^ ^]^ and CarSitePred^[^ ^11^ ^]^ on this dataset using a 10‐fold cross‐validation strategy. The results are summarized in Table S3 (Supporting Information), where we provide a comparison of our design with the other methods. The predictive performance of the competing methods is sourced from CarSitePred. We establish a threshold at which our model achieves the highest MCC values. SCANS records the max MCC values ranging from 0.712 to 0.779 across the four datasets. In comparison to the second‐best method CarSitePred, SCANS improves the predictive performance by 2.8% (0.764/0.743 = 1.028) to 5.8% (0.712/0.673 = 1.058). Table S3 (Supporting Information) also shows that the specificity and sensitivity values for SCANS exceed 0.9 and 0.8, respectively.

Table 3 presents a comparison of SCANS with the current methods on our newly compiled comprehensive benchmark testing dataset. SCANS achieves the average AUROC values of 0.823–0.900, demonstrating improvements of ≈31% (0.823/0.627 = 1.31, *p*‐value < 0.001) to 44% (0.885/0.614 = 1.44, *p*‐value < 0.001) over the second best iCarPS. When the specificity is set to 95% or 90%, SCANS maintains a TPR that is about 3.97–8.74 times its FPR across the four testing datasets. The corresponding ROC curves (Figure S6, Supporting Information) illustrate a substantial margin between the SCANS curve and those of other methods on the K carbonylation testing dataset. Particularly, we observe a sharp increase in the lower left part (0 < FPR < 0.1) of the ROC curves of SCANS. This indicates that even at thresholds where most nonfunctional residues are accurately identified, SCANS still successfully predicts a significant number of carbonylation sites. This observation is further supported by the robust AULCratio values, which all exceed 10 and even reach 27. The AULCratio measures the extent to which the AUROC value of a given predictor exceeds that of random predictions for low false‐positive rate scenarios (the left side of the ROC curve). In contrast, the AULCratio for other predictor does not exceed 4. The MCC and F1 are two comprehensive metrics that account for both true positives and true negatives. As they are considered balanced metrics, they provide accurate assessments of predictive performance, particularly for imbalanced data. To evaluate the optimal performance of each method, we calculate the maximum MCC values and maximum F1 scores for all methods. Table 3 demonstrates that SCANS yields commendable MCCmax > 0.33 and F1max > 0.37 across four carbonylation testing datasets, significantly surpassing the other methods (*p*‐value < 0.001). Figure S7 (Supporting Information) displays the PR curves of considered methods.

### 3.5 Assessment of the Cross‐ and Overpredictions

In proteins, not all lysine, proline, arginine, and threonine can undergo carbonylation. Actually, the vast majority of these four amino acids do not process specific functions; only a small fraction interacts with various ligands, while a select few may serve as carbonylation sites. This research is the first to consider the influence of ligand interaction sites, aiming to minimize cross‐prediction errors and develop a more accurate predictor compared to previous studies.^[^ ^11^, ^12^, ^13^, ^14^, ^15^, ^16^, ^17^, ^18^, ^21^ ^]^ Drawing inspiration from related studies,^[^ ^25^, ^28^ ^]^ we evaluate the cross‐predictions and overpredictions with the AUCPC and AUOPC. These two metrics quantify the extent to which ligand interaction sites and nonfunctional residues are misidentified as carbonylation sites. Lower values of AUCPC and AUOPC mean fewer cross‐predictions and overpredictions, respectively. To ease interpretation, we calculate the CPRratio and OPRratio, which represent the ratio of cross/overpredictions and sensitivity. A random‐level prediction gives ratio = 1, with higher scores indicating better performance relative to the random baseline.

Due to incomplete ligand interaction data in Jia's 250 proteins, we are unable to accurately determine whether these methods specifically identify carbonylation sites or inadvertently cross‐predict ligand interaction sites. Therefore, we estimate the false positive rates on our newly compiled benchmark testing datasets. As shown in **Table** **4**, SCANS achieves AUCPC values of 0.118, 0.067, 0.055, and 0.060 for the K, P, R, and T carbonylation testing datasets, respectively. In contrast, the AUCPC values for the other four methods range from 0.413 to 0.539. Although ≈90%–94% of the samples in the benchmark testing datasets are nonfunctional residues, SCANS exhibits significant reductions in AUOPC values. Specifically, compared to the second‐best iCarPS, SCANS reduces AUOPC values from 0.370 to 0.181 (*p* = 1.1E‐18) for the K carbonylation testing dataset, from 0.382 to 0.117 (*p* = 3.2E‐17) for the P carbonylation testing dataset, from 0.363 to 0.104 (*p* = 1.8E‐14) for the R carbonylation testing dataset, and from 0.367 to 0.123 (*p* = 4.8E‐14) for the T carbonylation testing dataset. The CPRratio and OPRratio metrics further highlight SCANS's superiority across the four datasets, a finding corroborated by the CP and OP curves shown in Figures S8 and S9 (Supporting Information). The SCANS curves are distinctly lower, illustrating a clear separation from the other methods.

**Table 4:** Assessment of cross predictions generated by SCANS compared to four current methods. Higher ratios (including CPRratio and OPRratio) and lower areas (including AUOPC and AUCPC) indicate more accurate predictions. We evaluate robustness across different datasets by conducting 10 tests on randomly selected 50% of the test dataset. We report the corresponding averages ± standard deviations, and *p*‐values between SCANS and each of the methods. The best results are highlighted in bold font.

Carbonylation type	Method	AUCPC	AUOPC	CPRratio		OPRratio	
				SP = 95%	SP = 90%	SP = 95%	SP = 90%
Lysine (K)	CarSPred	0.531 ± 0.015	0.514 ± 0.015	0.703 ± 0.166	0.898 ± 0.184	0.804 ± 0.131	0.990 ± 0.147
		*p* = 1.5E‐23	*p* = 6.4E‐22	*p* = 7.6E‐07	*p* = 2.3E‐06	*p* = 3.4E‐21	*p* = 5.8E‐22
	CarSPred‐2.0	0.461 ± 0.023	0.473 ± 0.021	1.971 ± 0.794	1.513 ± 0.262	1.433 ± 0.373	1.424 ± 0.206
		*p* = 1.9E‐19	*p* = 1.0E‐18	*p* = 8.3E‐07	*p* = 2.7E‐06	*p* = 7.7E‐20	*p* = 1.3E‐20
	iCarPS	0.413 ± 0.016	0.370 ± 0.011	1.104 ± 0.269	1.386 ± 0.151	1.136 ± 0.254	1.339 ± 0.166
		*p* = 2.6E‐20	*p* = 1.1E‐18	*p* = 7.8E‐07	*p* = 2.6E‐06	*p* = 1.4E‐20	*p* = 2.9E‐21
	CarSitePred	0.497 ± 0.015	0.470 ± 0.014	0.659 ± 0.228	0.592 ± 0.153	0.965 ± 0.253	0.779 ± 0.186
		*p* = 8.7E‐23	*p* = 7.7E‐21	*p* = 7.6E‐07	*p* = 2.1E‐06	*p* = 1.1E‐20	*p* = 9.1E‐22
	Motif‐based	0.404 ± 0.013	0.420 ± 0.010	4.498 ± 1.220	3.222 ± 0.529	2.086 ± 0.449	2.302 ± 0.180
		*p* = 5.9E‐21	*p* = 8.6E‐20	*p* = 1.6E‐07	*p* = 2.0E‐10	*p* = 6.9E‐18	*p* = 1.3E‐17
	**SCANS**	**0.118 ± 0.011**	**0.181 ± 0.011**	**196.428 ± 83.892**	**53.681 ± 24.539**	**12.15 ± 0.667**	**6.842 ± 0.282**
Proline (P)	CarSPred	0.476 ± 0.016	0.490 ± 0.021	3.408 ± 1.447	2.254 ± 0.544	1.355 ± 0.469	1.166 ± 0.313
		*p* = 2.4E‐21	*p* = 1.5E‐19	*p* = 1.0E‐04	*p* = 9.1E‐08	*p* = 2.5E‐16	*p* = 1.6E‐17
	CarSPred‐2.0	0.539 ± 0.025	0.490 ± 0.021	0.738 ± 0.448	0.804 ± 0.265	1.905 ± 2.582	1.188 ± 0.371
		*p* = 2.0E‐20	*p* = 1.1E‐19	*p* = 3.7E‐05	*p* = 3.2E‐08	*p* = 2.0E‐03	*p* = 4.1E‐17
	iCarPS	0.487 ± 0.031	0.382 ± 0.020	2.794 ± 1.054	1.467 ± 0.458	2.021 ± 0.429	1.367 ± 0.205
		*p* = 1.9E‐18	*p* = 3.2E‐17	*p* = 7.9E‐05	*p* = 5.1E‐08	*p* = 5.9E‐16	*p* = 7.3E‐18
	CarSitePred	0.447 ± 0.032	0.444 ± 0.022	0.827 ± 0.230	1.577 ± 0.438	1.573 ± 0.519	1.577 ± 0.303
		*p* = 1.6E‐17	*p* = 2.5E‐18	*p* = 3.8E‐05	*p* = 5.5E‐08	*p* = 4.5E‐16	*p* = 4.5E‐17
	Motif‐based	0.379 ± 0.034	0.397 ± 0.026	4.946 ± 2.649	3.775 ± 1.432	3.102 ± 0.812	2.622 ± 0.522
		*p* = 9.6E‐16	*p* = 2.2E‐16	*p* = 1.3E‐04	*p* = 5.1E‐04	*p* = 1.1E‐11	*p* = 1.7E‐10
	**SCANS**	**0.067 ± 0.018**	**0.117 ± 0.018**	**33.105 ± 18.863**	**22.616 ± 7.495**	**13.035 ± 1.227**	**7.672 ± 0.543**
Arginine (R)	CarSPred	0.427 ± 0.038	0.438 ± 0.027	4.279 ± 1.853	2.653 ± 0.682	3.098 ± 0.488	2.316 ± 0.441
		*p* = 1.8E‐16	*p* = 1.2E‐16	*p* = 1.1E‐05	*p* = 8.4E‐05	*p* = 5.9E‐12	*p* = 6.2E‐07
	CarSPred‐2.0	0.491 ± 0.022	0.489 ± 0.019	1.969 ± 0.725	1.002 ± 0.146	3.934 ± 1.668	1.167 ± 0.200
		*p* = 6.3E‐21	*p* = 3.6E‐19	*p* = 7.7E‐06	*p* = 5.9E‐05	*p* = 1.5E‐10	*p* = 8.9E‐08
	iCarPS	0.471 ± 0.035	0.363 ± 0.029	1.781 ± 0.552	1.695 ± 0.373	2.396 ± 0.674	2.225 ± 0.331
		*p* = 7.0E‐18	*p* = 1.8E‐14	*p* = 7.4E‐06	*p* = 6.9E‐05	*p* = 3.3E‐12	*p* = 5.1E‐07
	CarSitePred	0.532 ± 0.024	0.509 ± 0.025	0.572 ± 0.176	0.889 ± 0.186	0.830 ± 0.273	1.133 ± 0.196
		*p* = 3.2E‐21	*p* = 1.7E‐18	*p* = 6.3E‐06	*p* = 5.8E‐05	*p* = 4.1E‐13	*p* = 8.4E‐08
	Motif‐based	0.387 ± 0.019	0.397 ± 0.019	4.057 ± 0.827	4.063 ± 0.953	3.834 ± 0.545	2.834 ± 0.355
		*p* = 7.8E‐21	*p* = 2.5E‐18	*p* = 1.8E‐08	*p* = 1.8E‐07	*p* = 7.5E‐15	*p* = 8.1E‐08
	**SCANS**	**0.055 ± 0.015**	**0.104 ± 0.023**	**89.175 ± 44.543**	**55.565 ± 33.137**	**17.418 ± 2.840**	**10.561 ± 3.455**
Threonine (T)	CarSPred	0.502 ± 0.026	0.484 ± 0.021	1.972 ± 0.733	1.819 ± 0.651	1.857 ± 0.509	1.434 ± 0.271
		*p* = 2.3E‐20	*p* = 2.3E‐17	*p* = 2.4E‐05	*p* = 4.0E‐04	*p* = 5.4E‐16	*p* = 1.4E‐08
	CarSPred‐2.0	0.442 ± 0.028	0.460 ± 0.024	3.224 ± 1.517	2.133 ± 0.666	3.123 ± 0.671	2.113 ± 0.317
		*p* = 9.7E‐19	*p* = 2.2E‐16	*p* = 3.1E‐05	*p* = 4.3E‐04	*p* = 4.7E‐15	*p* = 5.0E‐08
	iCarPS	0.491 ± 0.030	0.367 ± 0.024	2.206 ± 0.770	2.090 ± 0.447	1.595 ± 0.245	1.944 ± 0.290
		*p* = 2.9E‐19	*p* = 4.8E‐14	*p* = 2.5E‐05	*p* = 4.2E‐04	*p* = 2.1E‐16	*p* = 3.6E‐08
	CarSitePred	0.534 ± 0.044	0.533 ± 0.033	1.320 ± 0.559	1.287 ± 0.613	1.346 ± 0.465	1.154 ± 0.259
		*p* = 2.1E‐17	*p* = 9.0E‐17	*p* = 2.1E‐05	*p* = 3.5E‐04	*p* = 2.5E‐16	*p* = 8.6E‐09
	Motif‐based	0.380 ± 0.024	0.388 ± 0.022	14.177 ± 11.470	5.250 ± 1.804	5.145 ± 1.043	4.007 ± 0.959
		*p* = 1.6E‐18	*p* = 1.2E‐16	*p* = 6.6E‐04	*p* = 8.2E‐08	*p* = 7.2E‐13	*p* = 2.2E‐07
	**SCANS**	**0.060 ± 0.014**	**0.123 ± 0.028**	**60.806 ± 33.019**	**41.105 ± 28.645**	**16.265 ± 1.613**	**10.035 ± 2.788**

### 3.6 Interpretation of SCANS Model and Ablation Analysis

SCANS first pretrains the LISPM and then freezes the pretrained networks when subsequently incorporating the CSPM. Both modules feed CNN with physicochemical properties and TRF with evolutionary profiles to capture the local features and global features, respectively. The CNN block emphasizes meaningful features along two dimensions: channel and spatial axes. It is designed to facilitate efficient information flow within the network by identifying which features should be suppressed and which should be emphasized. The TRF block highlights the benefits of long distance evolution‐based features, which excel at capturing global relationships and thereby overcome some limitations of the CNN block.

To verify the contributions of the four innovations to the predictive performance, we conduct an ablation analysis where we remove each innovation. This leads to the following four configurations: 1) excluding ESM2 encodings, which removes ESM2 encodings from the CSPM; 2) replacing the long and short distance attention mechanism units, where the CNN and TRF units in both the CSPM and LISPM are replaced by FCNN units; 3) removing transfer learning, which we retain the entire networks and do not pretrain the LISPM; and 4) using common loss functions, where we re‐train the networks with commonly used loss functions.

**Figure 4:**
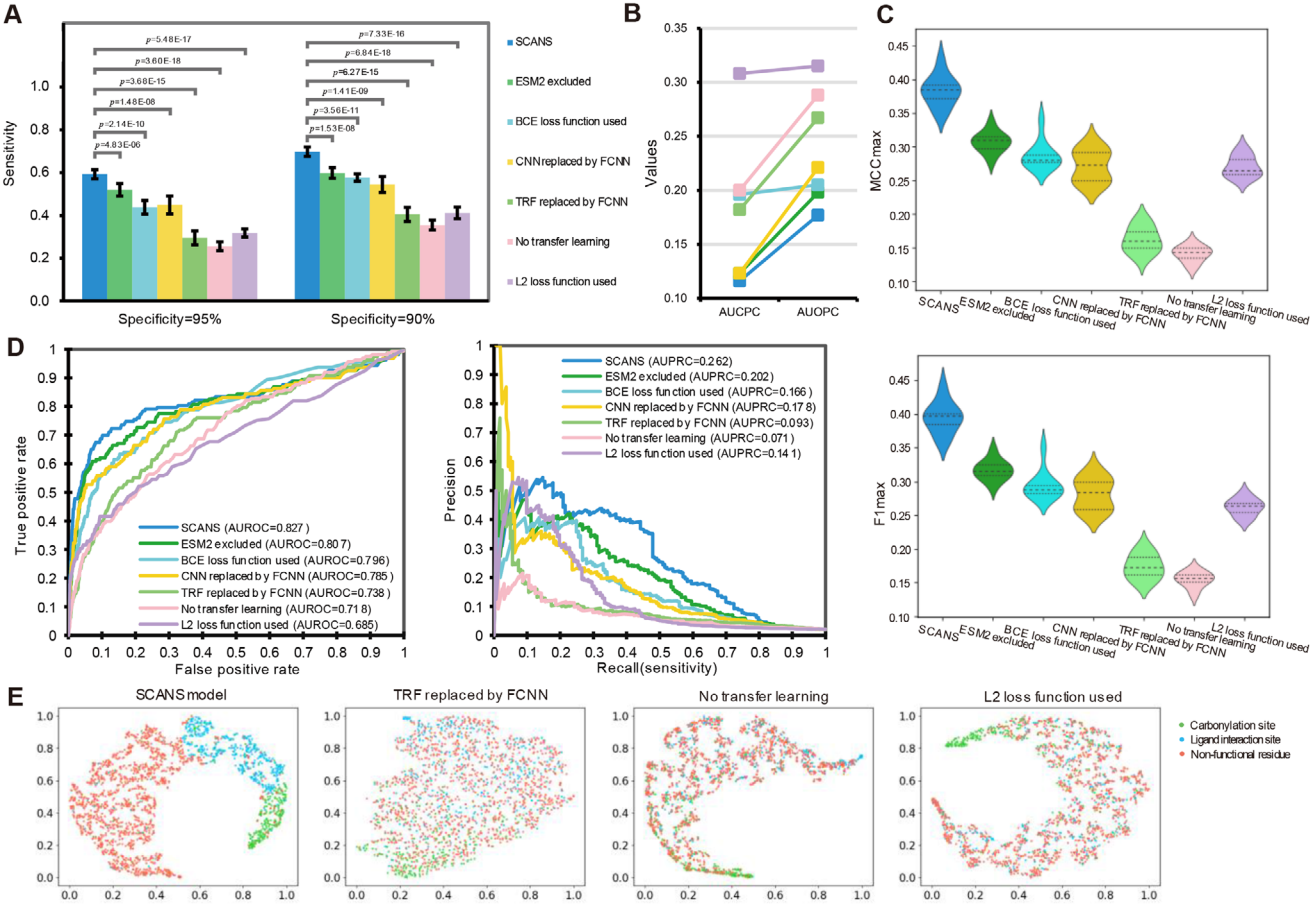
Ablation analysis on the test dataset that compares SCANS with its variants where one of the four innovations is removed. A) The bar chart of sensitivities at 5% and 10% FPR for SCANS and the other ablation setups. B) The overall cross‐prediction and overprediction results. C) The violin plots of the MCCmax and F1max values. D) The ROC and PR curves of SCANS and different variants. E) The t‐SNE clustering results of the output features of SCANS and three ablation setups.

**Figure** **4** illustrates and compares the prediction performance of the SCANS model on the K test data with four ablation setups. We notice that each innovation significantly improves sensitivity (*p*‐value < 0.001) compared to the complete SCANS network (Figure 4A). This is further supported by Figure 4D, which shows above all ablation models. Figure 4B summarizes the cross‐predictions and overpredictions errors, where lower values indicate better performance and higher represent the opposite. Notably, the model that excludes ESM2 yields the second highest sensitivity, AUROC and AUPRC values, indicating that ESM2 contributes less compared to other innovations. The three most significant drops in AUROC values occur when TRF is replaced with FCNN, transfer learning is removed, and L2 loss is used in place of our customized loss function. Furthermore, when employing common L2 or Binary Cross Entropy (BCE) loss function, both AUCPC and AUOPC values increase significantly. This suggests that our customized loss function effectively optimizes the model to focus more on the identification of carbonylation sites. The introduction of the LISPM, along with transfer learning, effectively penalizes cross‐prediction errors. The violin plots in Figure 4C depict the results of MCCmax and F1max, respectively. The exclusion of transfer learning and the replacement of TRF with FCNN result in the largest declines in both MCCmax and F1max values. This is consistent with Figure 4D. The PR curves of these two ablation setups are positioned at the bottom among all curves. Based on the impact of different ablation setups on the model, we select the three setups: remove transfer learning, replace the customized loss function with L2 loss function, and substituting TRF with FCNN. Figure 4E exhibits the clustering results obtained through t‐SNE for the SCANS method, along with those for three ablation setups. It is clear to see that these configurations assist in separating the green points (carbonylation sites), blue points (ligand interaction sites), and the majority red points (nonfunctional residues). Figures S10–S13 (Supporting Information) displays the SCANS model ablation setups on P, R, T carbonylation testing datasets.

### 3.7 Comparative Assessment of Cross‐Predictions on Ligand Interaction Dataset

In Section 3.5, we observe that the current predictors all generate a considerable number of false positives, particularly in cross‐predictions. To further assess the validity of these methods, we conduct experiments using a specialized dataset that excludes carbonylation sites and includes only ligand interaction sites. Ideally, an accurate model should achieve a false positive rate (or CPR) of zero. We posit that methods recognizing fewer carbonylation sites (indicating a lower CPR) exhibit superior specificity. We randomly select 100 ligand interaction proteins from BioLiP2. As detailed in Section 2.1, these proteins possess full UniProt sequences and complete ligand interaction data. We further ensure these proteins share no more than 25% sequence similarity with our training dataset using Blastclust. Consequently, we identified 111 lysine (K), 58 proline (P), 144 arginine (R), and 134 threonine (T) ligand interaction segments. These segments are subsequently utilized to evaluate the performance of CarSitePred, CarSPred, CarSPred‐2.0, iCarPS, and our SCANS. We establish thresholds at SP = 95% and 90% to compare the false positives (cross‐predictions) of various methods across four types of ligand interaction datasets. Therefore, a random result would consequently yield 5% and 10% false positives, respectively.

**Figure 5:**
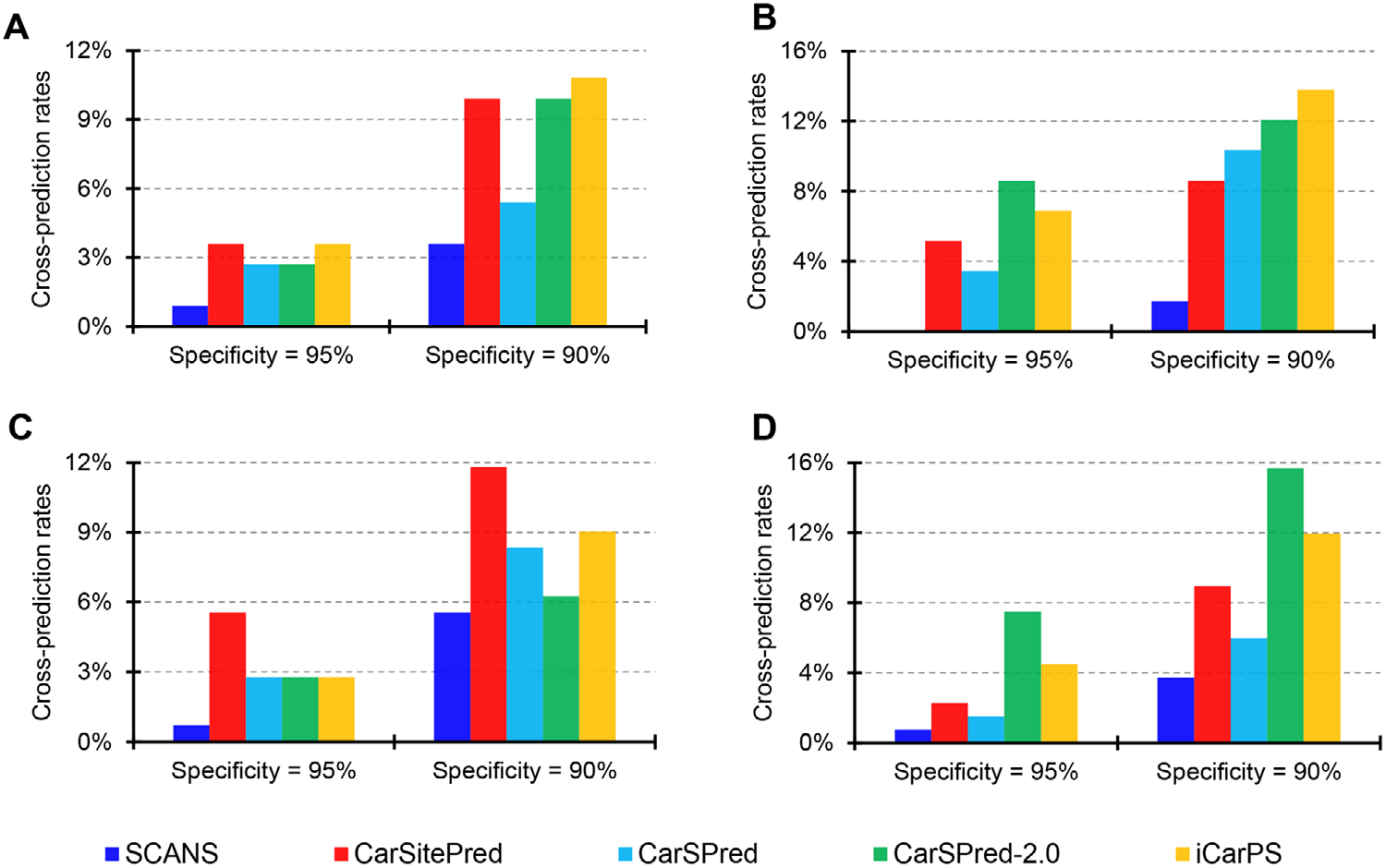
Comparison of cross‐predictions among various methods on the ligand interaction datasets for K (panel A), P (panel B), R (panel C), and T (panel D).

As illustrated in **Figure** **5**, CarSitePred cross‐predicts more than 4% of K, P, and R ligand interaction sites, and 2% T ligand interaction sites as carbonylation sites when the threshold is set at SP = 95%. This fraction increases to 8∼12% at SP = 90%. CarSPred demonstrates slightly better on K and T ligand interaction datasets, while CarSPred‐2.0 and iCarPS exhibit poorer performance on K, P, and T datasets. In contrast to other predictors, the proposed SCANS achieves the fewest false positives, with less than 1% and 4% CPR at the two thresholds, respectively (the only exception being an CPR of 5.6% at SP = 90% for the R ligand interaction sites dataset). Particularly, SCANS attains an CPR = 0 at SP = 95% for the P ligand interaction sites dataset. This superior performance can be attributed to SCANS being trained on a dataset containing diverse types of ligand interaction sites, thereby enhancing its ability to accurately mitigate the influence of these sites.

### 3.8 Application of SCANS on human carbonylated proteins

We apply the proposed SCANS to predict human carbonylated proteins. Specifically, we first exclude the human carbonylated segments from our training dataset and then proceed to train the model. This way, we eliminate the optimization bias during the training process. We empirically use the optimal parameters of window size and customized loss function. Finally, we visually evaluate our method against the current best predictor iCarPS, and the motif‐based model on human carbonylated proteins.

**Figure 6:**
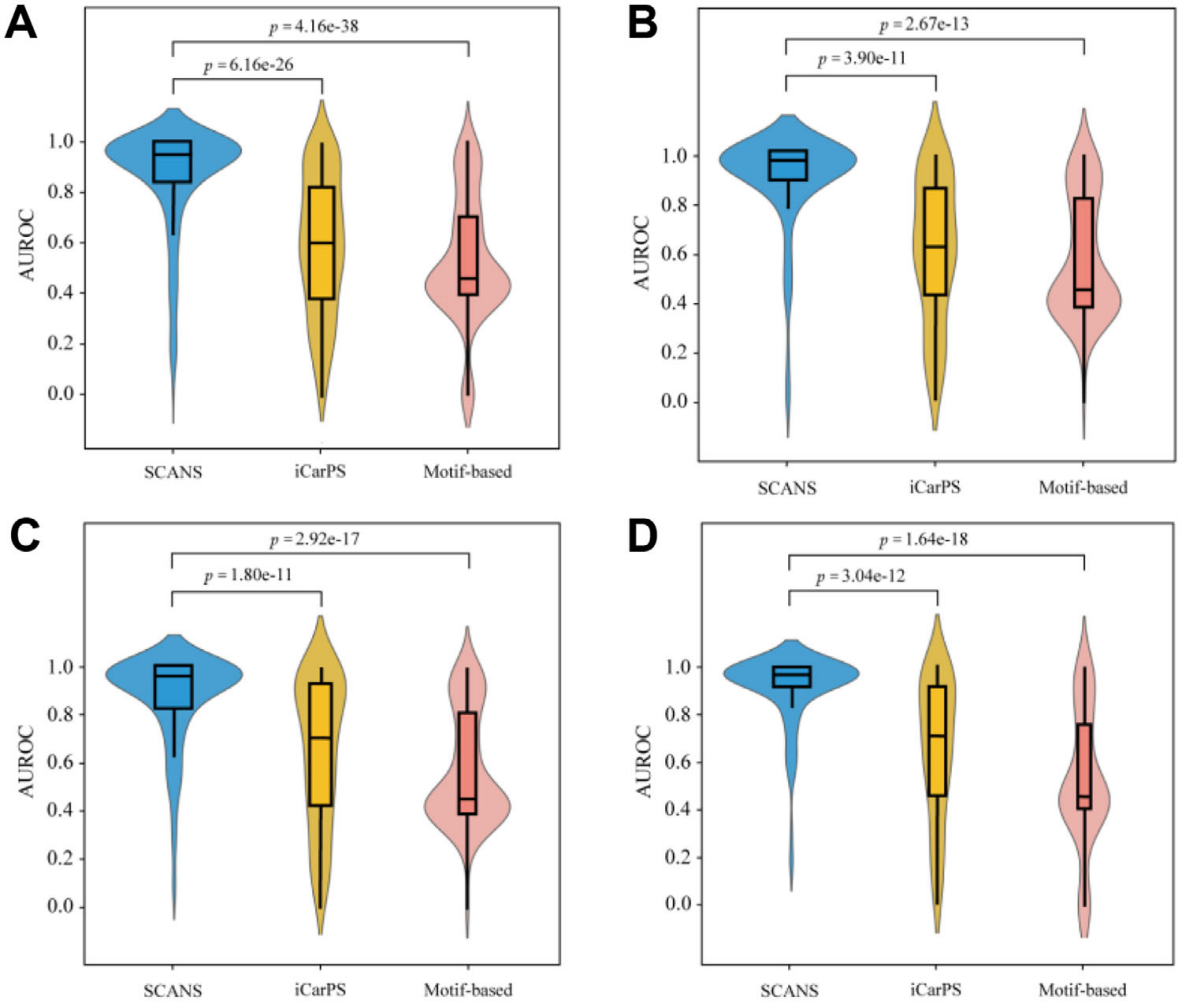
The violin plots of AUROC values of SCANS, iCarPS, and Motif‐based methods on human K (panel A), P (panel B), R (panel C), and T (panel D) carbonylated proteins.

**Figure** **6** illustrates the distribution of AUROC values for human K, P, R, and T carbonylated proteins. In these violin plots, wider sections indicate a higher density of AUROC scores, while narrower ones reflect a lower density. Generally, the SCANS method shows a relatively high density of AUROC values ranging from 0.8 to 1.0. The median AUROC values for the K, P, R, and T carbonylated proteins are 0.952, 0.979, 0.966, and 0.974, respectively. In contrast, the AUROC values obtained from the iCarPS predictor exhibit an approximately normal distribution, with median values of ≈0.606 (*p* = 6.16E‐26), 0.636 (*p* = 3.90E‐11), 0.713 (*p* = 1.80E‐11), and 0.706 (*p* = 3.04E‐12). The motif‐based models display two wide sections; their overall AUROC values are the lowest among the considered three methods. These results demonstrate that the proposed SCANS promises good performance in identifying carbonylation sites in human carbonylated proteins.

## 4 Conclusion

Understanding protein carbonylation is crucial in molecular biology and medicine, as it plays a significant role in the pathophysiology of various human diseases and the aging process. While numerous methods have been developed to predict carbonylation sites, many of them tend to make excessive cross‐predictions for ligand interaction sites rather than specifically identifying carbonylation sites. We address this gap by introducing SCANS, a novel accurate tool that makes predictions of carbonylation sites based on protein sequences. SCANS relies on a sophisticated interpretable deep network model that implements several innovations including the use of multilevel attention strategy, transfer learning, multistep architecture, and a custom‐designed loss function. We conduct a detailed analysis of these innovations and empirically validate their effectiveness. Independent tests on low‐similarity test datasets reveal that SCANS produces accurate predictions of carbonylation sites with a low rate of cross‐predictions. Ablation studies demonstrate that each of the aforementioned innovations facilitates a substantial boost to the predictive performance. Using a newly compiled independent testing dataset comprising carbonylation sites and various ligand interaction sites, we also show that SCANS consistently produces relatively low rates of cross‐predictions and overpredictions. Moreover, we employ information theory‐based approach to obtain computationally derived carbonyl‐related motifs, which we subsequently analyze and use to develop corresponding models. We share the data, code and results of SCANS in the GitHub repository (https://github.com/jianzhang‐xynu/SCANS).

## Conflict of Interest

The authors declare no conflict of interest.

## Supporting information



Supporting Information

## Data Availability

The authors declare that the data supporting the findings of this study are available within the article and its supplementary information files. The source code, benchmark datasets, final models and results are all available at the GitHub (https://github.com/jianzhang‐xynu/SCANS). We detail the experimental settings and tutorial of the predictor in the GitHub repository.
