# Supplementary material for: Explainable Deep Multilevel Attention Learning for Predicting Protein Carbonylation Sites
Source: Adv Sci (Weinh). 2025 Mar 27;12(23):2500581. doi: 10.1002/advs.202500581 (PMC12199415; doi:10.1002/advs.202500581)
Supplement: Supplementary file 1 — Supporting Information [file ADVS-12-2500581-s001.docx]

Supporting Information

Explainable Deep Multi-level Attention Learning for Predicting Protein Carbonylation Sites

Jian Zhang^1,2,*^, Jingjing Qian^1,2^, Pei Wang^3^, Xuan Liu^1^, Fuhao Zhang^4^, Haiting Chai^5^, Quan Zou^2,*^

^1^School of Computer and Information Technology, Xinyang Normal University, Xinyang, 464000 China

^2^Yangtze Delta Region Institute (Quzhou), University of Electronic Science and Technology of China, Quzhou, 324003 China

^3^Department of Anesthesia, Critical Care and Pain Medicine, Massachusetts General Hospital, Harvard Medical School, Boston, MA, 02114 USA

^4^College of Information Engineering, Northwest A&F University, Yangling, Shaanxi, 712100 China

^5^Nuffield Department of Medicine, University of Oxford, Oxford, OX1 3SY, UK

*Corresponding author: jianzhang@xynu.edu.cn; zouquan@nclab.net

## Supplementary tables

**Table S1.** The breakdown of the physicochemical properties of 20 standard amino acids

| Amino acid Type | Hydrophilicity | Hydrophobicity | Tiny | Acidicity | Positively charged | Negatively charged | Polarity | Aromaticity | Sulphur content | Aliphatic |
| --- | --- | --- | --- | --- | --- | --- | --- | --- | --- | --- |
| A | 0 | 1 | 1 | 0 | 0 | 0 | 0 | 0 | 0 | 1 |
| C | 0 | 1 | 1 | 0 | 0 | 0 | 1 | 0 | 1 | 0 |
| D | 0 | 0 | 0 | 0 | 0 | 1 | 1 | 0 | 0 | 0 |
| E | 1 | 0 | 0 | 0 | 0 | 1 | 1 | 0 | 0 | 0 |
| F | 0 | 1 | 0 | 0 | 0 | 0 | 0 | 1 | 0 | 0 |
| G | 0 | 0 | 1 | 0 | 0 | 0 | 0 | 0 | 0 | 1 |
| H | 0 | 1 | 0 | 0 | 0 | 0 | 1 | 1 | 0 | 0 |
| I | 0 | 1 | 0 | 0 | 0 | 0 | 0 | 0 | 0 | 1 |
| K | 1 | 0 | 0 | 0 | 1 | 0 | 1 | 0 | 0 | 0 |
| L | 0 | 1 | 0 | 0 | 0 | 0 | 0 | 0 | 0 | 1 |
| M | 0 | 1 | 0 | 0 | 0 | 0 | 0 | 0 | 1 | 0 |
| N | 1 | 0 | 0 | 1 | 0 | 0 | 1 | 0 | 0 | 0 |
| P | 0 | 0 | 0 | 0 | 0 | 0 | 0 | 0 | 0 | 0 |
| Q | 0 | 0 | 0 | 1 | 0 | 0 | 1 | 0 | 0 | 0 |
| R | 0 | 0 | 0 | 0 | 1 | 0 | 1 | 0 | 0 | 0 |
| S | 1 | 0 | 1 | 0 | 0 | 0 | 1 | 0 | 0 | 0 |
| T | 0 | 1 | 0 | 0 | 0 | 0 | 1 | 0 | 0 | 0 |
| V | 0 | 1 | 0 | 0 | 0 | 0 | 0 | 0 | 0 | 1 |
| W | 0 | 1 | 0 | 0 | 0 | 0 | 1 | 1 | 0 | 0 |
| Y | 1 | 0 | 0 | 0 | 0 | 0 | 1 | 1 | 0 | 0 |

**Table S2**. The selective computation-based carbonylation-related motifs.

- Lysine carbonylation related motifs

| *LRTG, RKME, QTAL, KxKxLKS, KKQT, LxExLxQE, LxLKxKxExE, KxKxSxH, PKAT, KxQExF, ExKxQxKxA, KxKEKxE, LxSxKxPxE, KALV, ExQxKxLxExL, LxKTV, KxKxKExE, ExKDxExExK, EKxKxKxK, ExKxQxI, QxKxLxLxKxE, ExKxQxKxLxK, ExKDxE, IxKxKxAxL, KSKE, KExKxEKxK, EKxEKxK, LxAxLxKxK, ExIQ, QxKxKxT, NxQE, KxVxLxKxD, PxExKxQ, KxKIxE, DKxLxK, CxExKxL, KxKxNxK, SxTxKxK, KxExAxE* |
| --- |

- Proline carbonylation related motifs

| *ExKxPxE, DxKxK, PxVQ, VxNxPxK, ExSxN, ExExPxE, SxKxKxP, KxExExP, PxExQxK, SxKK, KxKxPxK, KxDxS, AxKxKxK* |
| --- |

- Arginine carbonylation related motifs

| *KxRxLxLxRxA, LRxRxPxR, LxRxLxLxRxS, LxRxRxK, IxRxQxE, RxKxQxExL, RxSxKxExL, LxRxCxA, VxSxRxRxR, QExExE, LxRxPxRxE, EKE, RTxR, LxRxLxQ, KxQV, ExExER, LxLxRxL, SxKE* |
| --- |

- Threonine carbonylation related motifs

| *KxKAxK, KEQ, PLK, KxKxTxLxK, KxSTxS, PAP, PxKxAxP, KxSxTxS, KxKxTxLxS, SxTxDxExL, GxKL, KExT, SxKE, KxSxSxS* |
| --- |

**Table S3**. Predictive performance of 10-fold cross-validation on Jia’s 250 proteins.

| Carbonylation Type | Method | SN | SP | ACC | MCC |
| --- | --- | --- | --- | --- | --- |
| Lysine (K) | PTMPred | 0.235 | 0.930 | 0.886 | 0.189 |
|  | CarSpred | 0.232 | 0.924 | 0.872 | 0.227 |
|  | iCar-PseCp | 0.452 | 0.993 | 0.844 | 0.591 |
|  | CarSite | 0.663 | 0.734 | 0.725 | 0.294 |
|  | CarSite-II | 0.852 | 0.819 | 0.827 | 0.607 |
|  | Carsite_AGan | 0.650 | 0.790 | N/A | N/A |
|  | CarSitePred | 0.857 | 0.865 | 0.861 | 0.723 |
|  | SCANS | 0.893 | 0.906 | 0.904 | 0.751 |
| Proline (P) | PTMPred | 0.214 | 0.932 | 0.829 | 0.257 |
|  | CarSpred | 0.253 | 0.933 | 0.829 | 0.233 |
|  | iCar-PseCp | 0.482 | 0.985 | 0.868 | 0.601 |
|  | CarSite | 0.706 | 0.737 | 0.733 | 0.328 |
|  | CarSite-II | 0.923 | 0.809 | 0.827 | 0.582 |
|  | Carsite_AGan | 0.680 | 0.830 | N/A | N/A |
|  | CarSitePred | 0.831 | 0.840 | 0.836 | 0.673 |
|  | SCANS | 0.868 | 0.897 | 0.892 | 0.712 |
| Arginine (R) | PTMPred | 0.200 | 0.910 | 0.866 | 0.188 |
|  | CarSpred | 0.255 | 0.934 | 0.862 | 0.225 |
|  | iCar-PseCp | 0.467 | 0.996 | 0.842 | 0.608 |
|  | CarSite | 0.655 | 0.660 | 0.659 | 0.225 |
|  | CarSite-II | 0.900 | 0.824 | 0.832 | 0.511 |
|  | Carsite_AGan | 0.690 | 0.820 | N/A | N/A |
|  | CarSitePred | 0.878 | 0.878 | 0.878 | 0.755 |
|  | SCANS | 0.895 | 0.936 | 0.929 | 0.779 |
| Threonine (T) | PTMPred | 0.224 | 0.914 | 0.884 | 0.219 |
|  | CarSpred | 0.214 | 0.934 | 0.866 | 0.204 |
|  | iCar-PseCp | 0.507 | 0.986 | 0.862 | 0.619 |
|  | CarSite | 0.683 | 0.736 | 0.728 | 0.323 |
|  | CarSite-II | 0.999 | 0.829 | 0.854 | 0.644 |
|  | Carsite_AGan | 0.650 | 0.730 | N/A | N/A |
|  | CarSitePred | 0.810 | 0.929 | 0.869 | 0.743 |
|  | SCANS | 0.811 | 0.958 | 0.934 | 0.764 |

## Supplementary figures

| 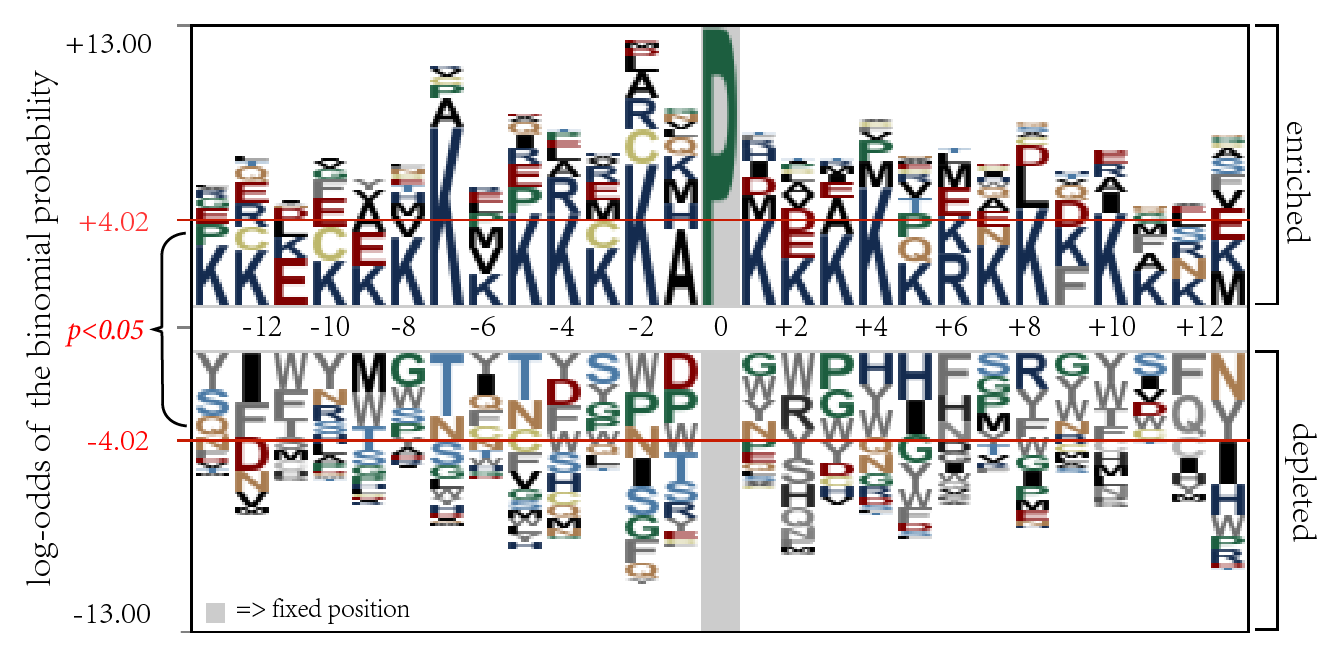  **A** |
| --- |
| 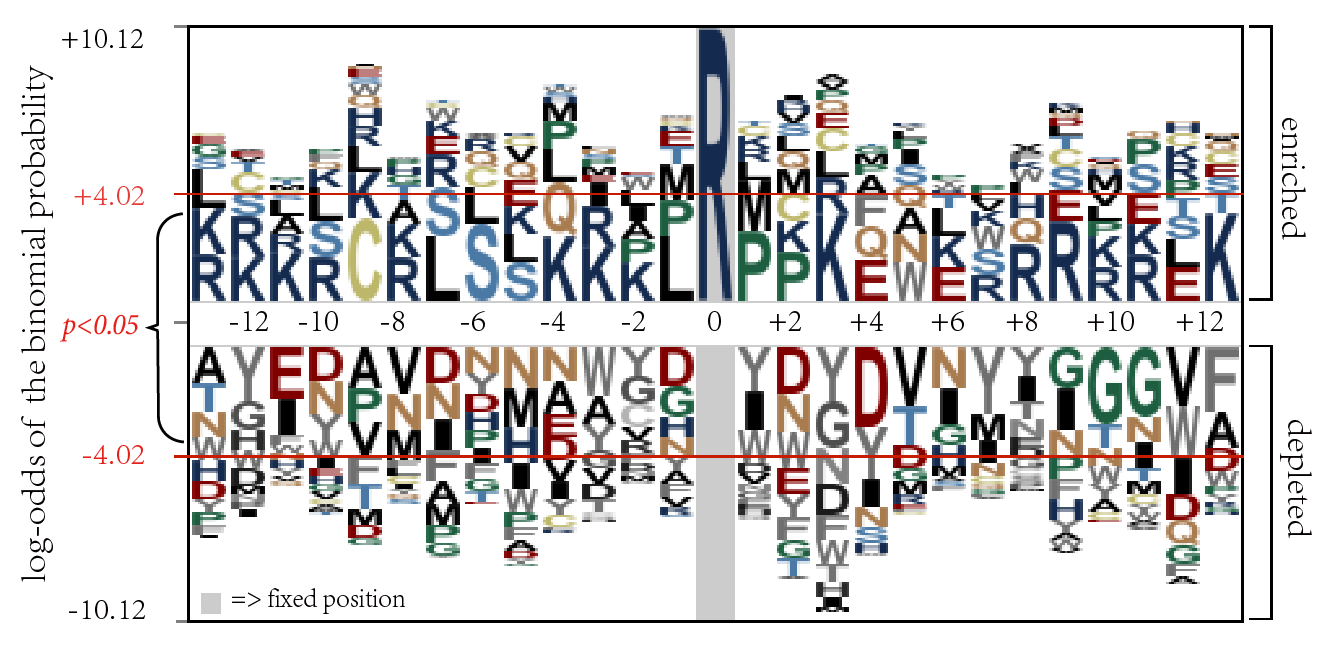  **B** |
| 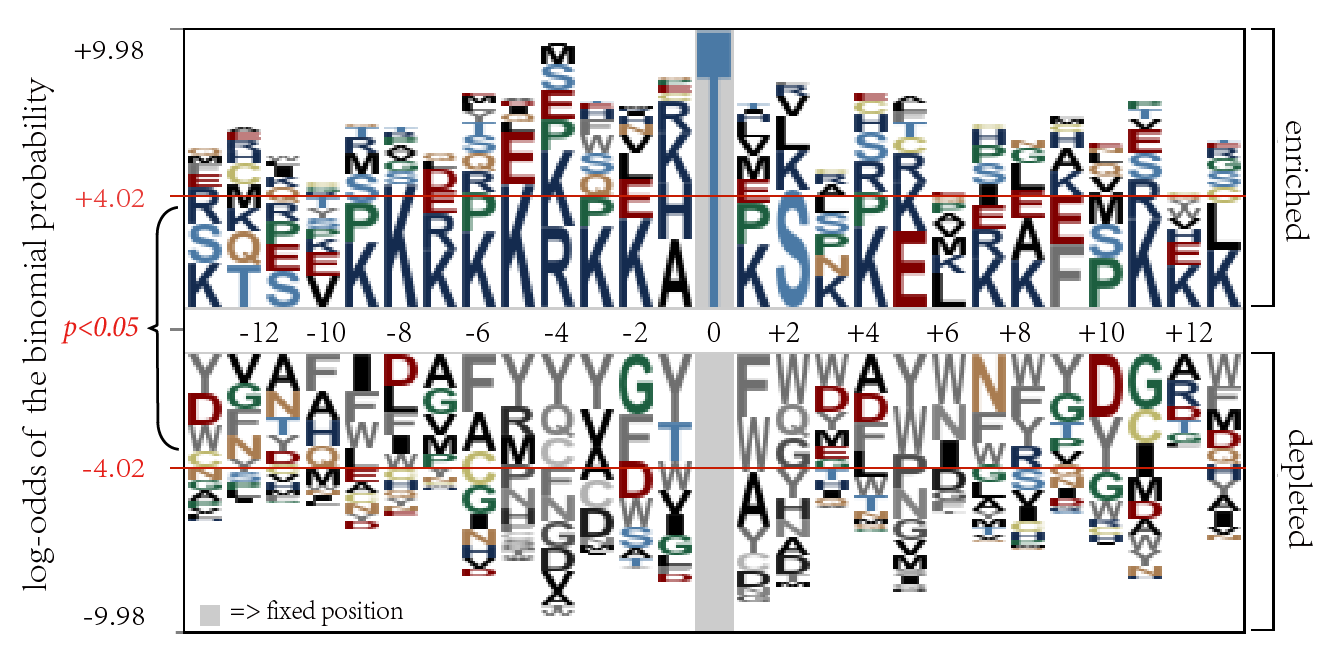  **C** |

**Figure S1**. Sequence logo of carbonylated segments vs. non-carbonylated segments. A) Sequence logo of proline. B) Sequence logo of arginine. C) Sequence logo of threonine.

| 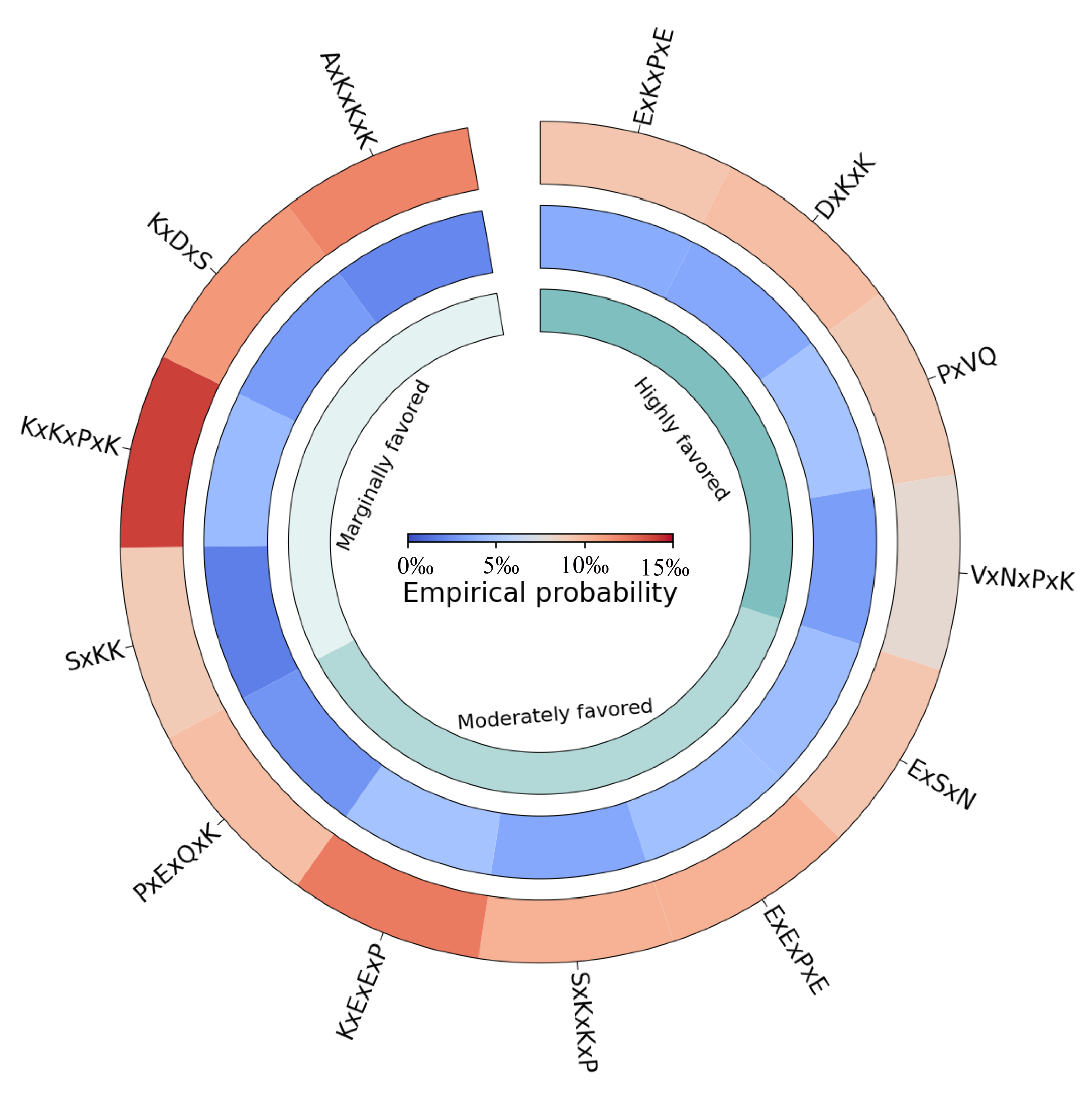  **A** | 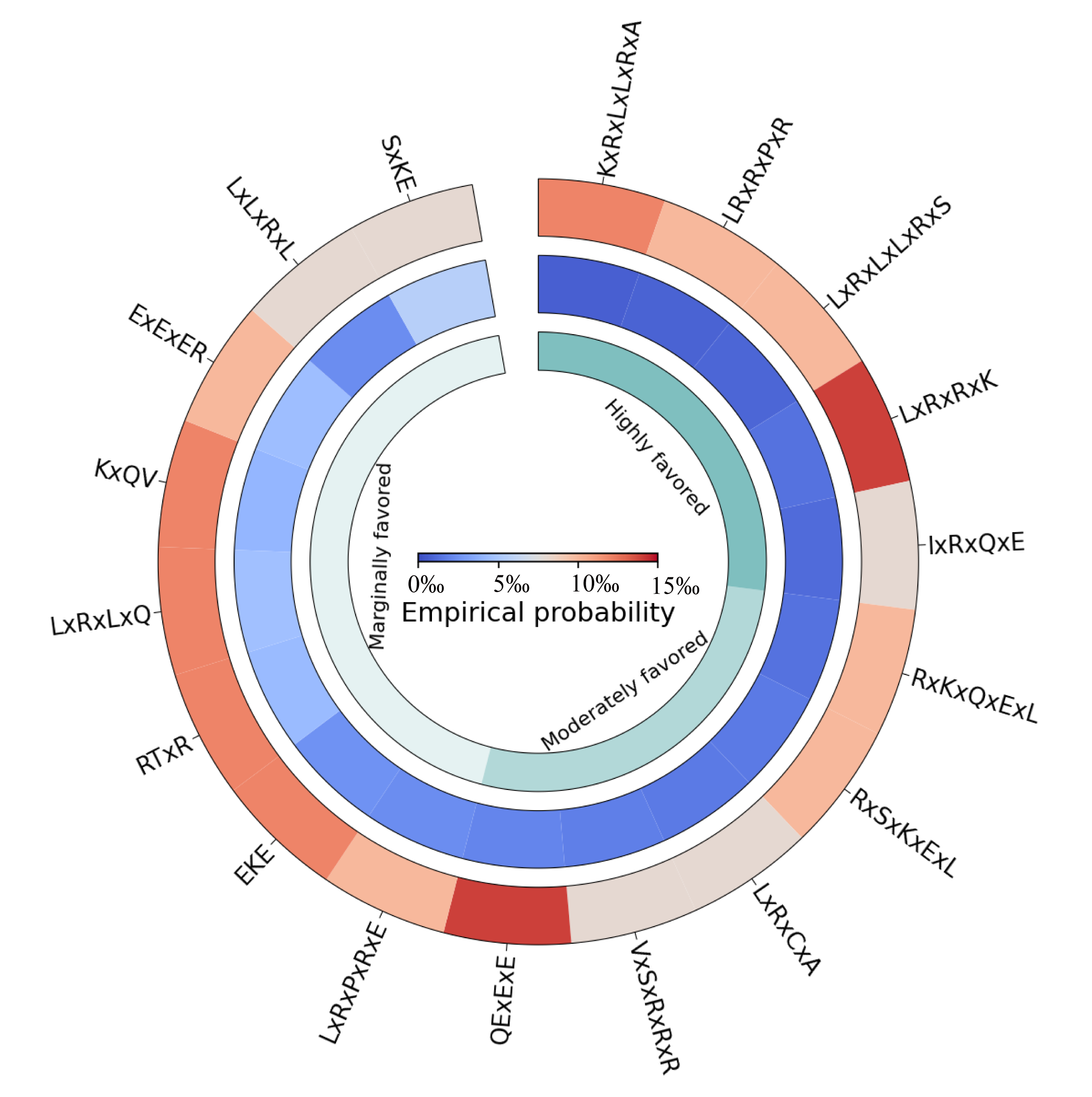  **B** |
| --- | --- |
| 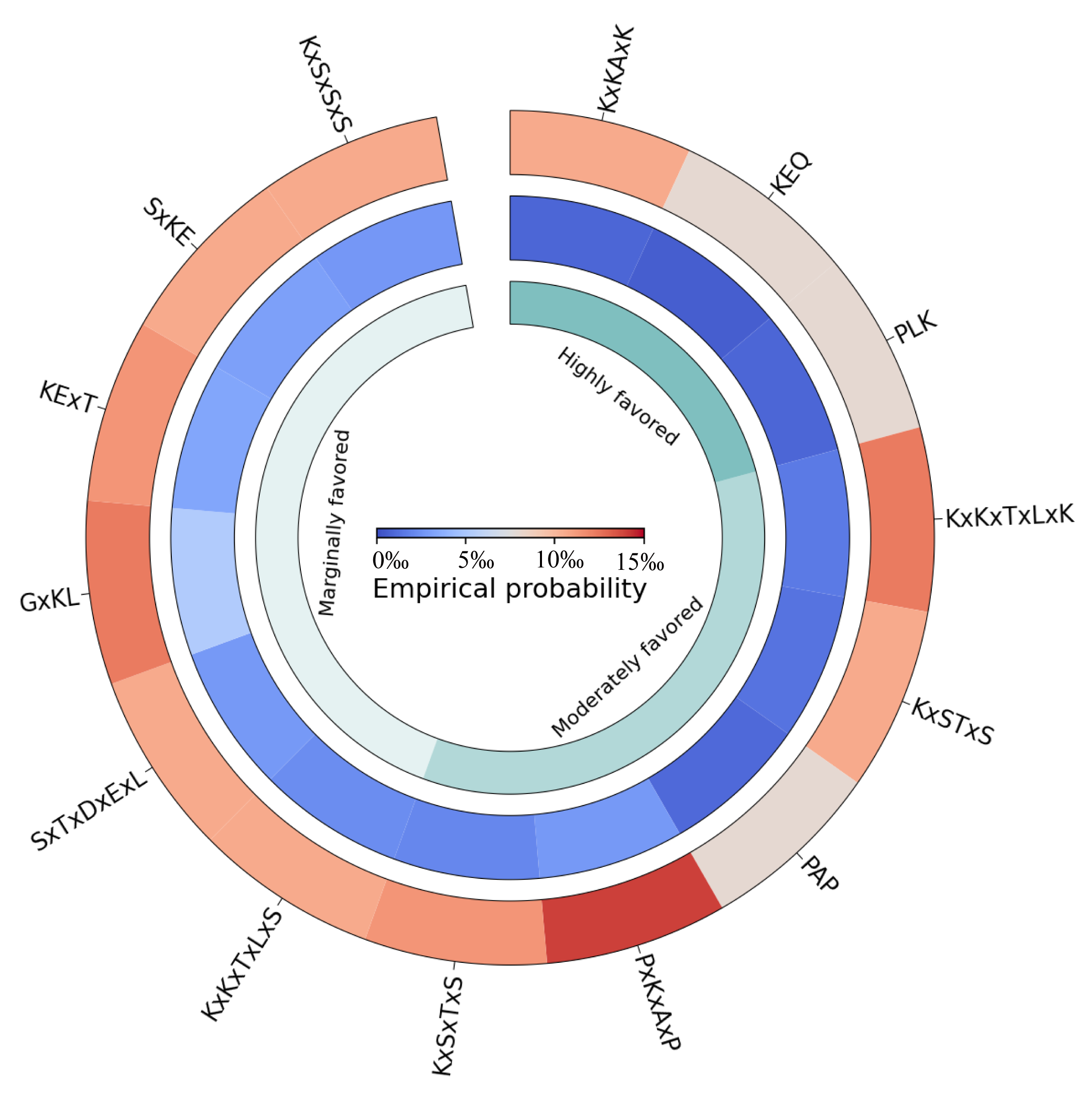  **C** |  |

**Figure S2**. The Circos heatmap of the empirical probabilities of selected motifs on carbonylation segments vs. non-carbonylation segments. A) Proline carbonylation segments vs. non-carbonylation segments. B) Arginine carbonylation segments vs. non-carbonylation segments. C) Threonine carbonylation segments vs. non-carbonylation segments.

| **A** | **B** |
| --- | --- |
| **C** | **D** |
| **E** | **F** |
|  | |

**Figure S3**. The ROC curves (provided for the FPR < 0.2) and PR curves (provided for the TPR < 0.2) of the motif-based model. A) The ROC curves for P carbonylation training and testing dataset. B) The PR curves for P carbonylation training and testing dataset. C) The ROC curves for R carbonylation training and testing dataset. D) The PR curves for R carbonylation training and testing dataset. E) The ROC curves for T carbonylation training and testing dataset. F) The PR curves for T carbonylation training and testing dataset.

| **A** 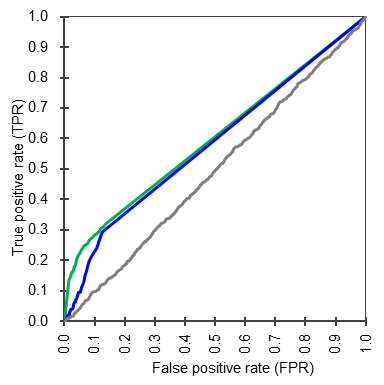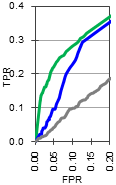 | **B** 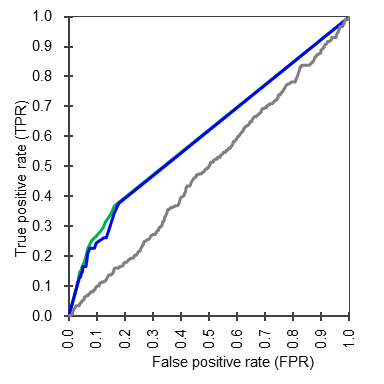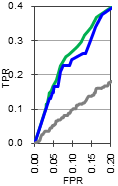 |
| --- | --- |
| **C** 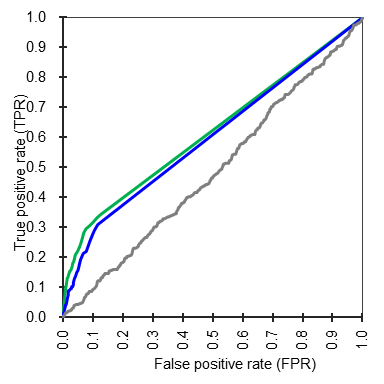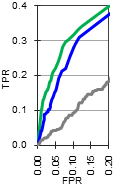 | **D** 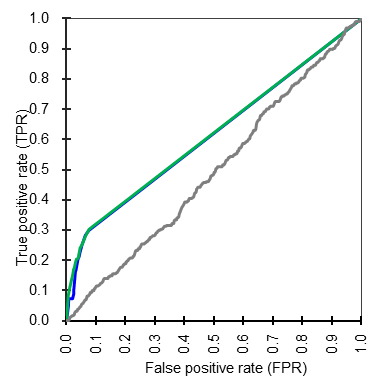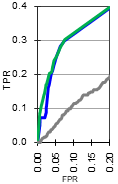 |
|  | |

**Figure S4**. The ROC curves of the motif-based model. A) The ROC curves for K carbonylation training and testing dataset. B) The ROC curves for P carbonylation training and testing dataset. C) The ROC curves for R carbonylation training and testing dataset. D) The ROC curves for T carbonylation training and testing dataset.

| **A** | **B** |
| --- | --- |
|  | **D** |

**Figure S5.** Prediction results of lysine K (panel A), proline P (panel B), arginine R (panel C), and threonine T (panel D) carbonylation validation datasets intercepted by different window sizes.

| **A** | **B** |
| --- | --- |
| **C** | **D** |

**Figure S6**. The ROC curves of different methods computed on the K (panel A), P (panel B), R (panel C), and T (panel D) carbonylation testing dataset.

| **A** | **B** |
| --- | --- |
| **C** | **D** |

**Figure S7**. The precision-recall curves of different methods computed on the K (panel A), P (panel B), R (panel C), and T (panel D) carbonylation testing dataset.

| **A** | **B** |
| --- | --- |
| **C** | **D** |

**Figure S8**. The cross-prediction curves of different methods computed on the K (panel A), P (panel B), R (panel C), and T (panel D) carbonylation testing dataset.

| **A** | **B** |
| --- | --- |
| **C** | **D** |

**Figure S9**. The over-prediction curves of different methods computed on the K (panel A), P (panel B), R (panel C), and T (panel D) carbonylation testing dataset.

| **A** | **B** |  |
| --- | --- | --- |
| **C** | **D** |  |
| **E** | **F** |  |

**Figure S10**. The bar chart of sensitivities at 95% and 90% specificity of SCANS and the other ablation setups on P (panel A), R (panel C), and T (panel E) carbonylation testing datasets. The overall cross-prediction and over-prediction results on P (panel B), R (panel D), and T (panel F) carbonylation testing datasets.

| **A** | **B** |
| --- | --- |
| **C** | **D** |
| **E** | **F** |

**Figure S11**. The cross-prediction curves computed on the P (panel A), R (panel C), and T (panel E) carbonylation testing datasets. The precision-recall curves computed on the P (panel B), R (panel D), and T (panel F) carbonylation testing datasets.

| 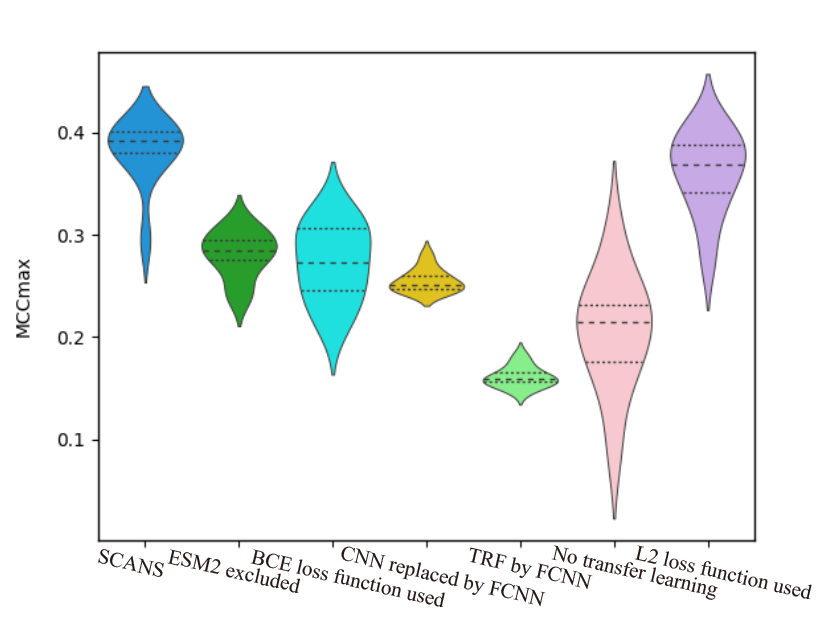  **A** | 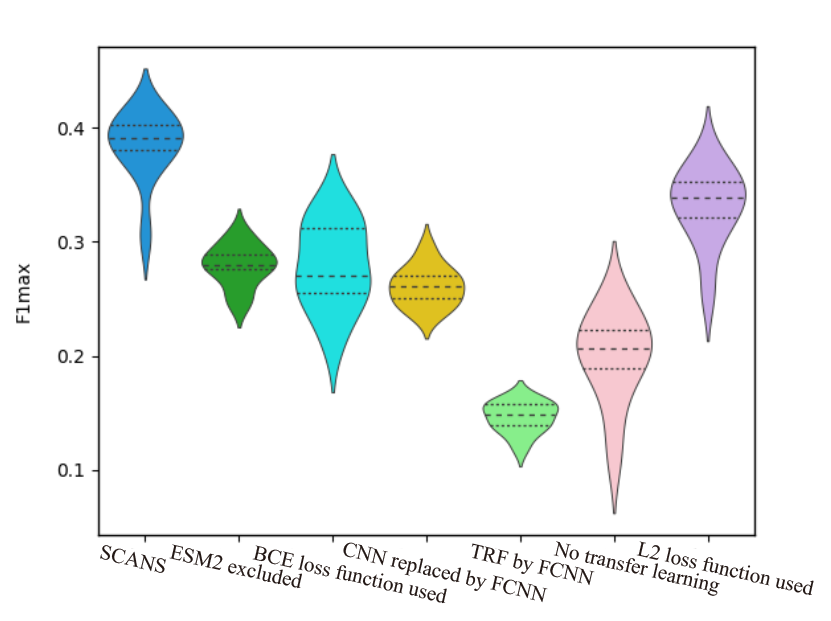  **B** |
| --- | --- |
| 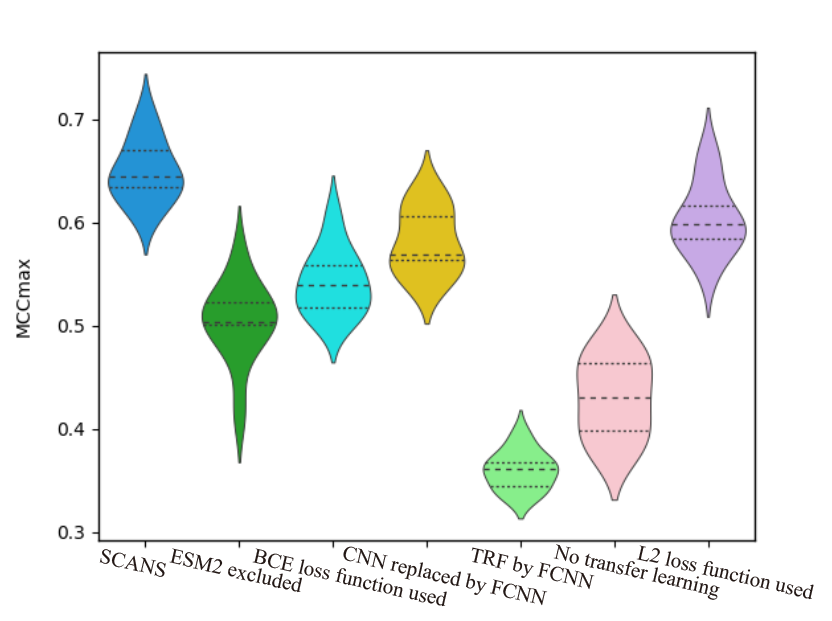  **C** | 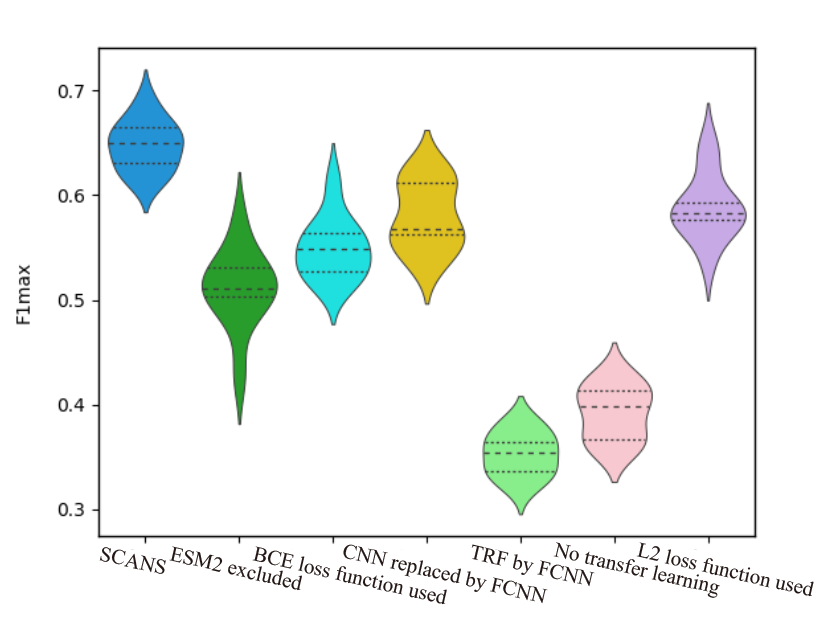  **D** |
| 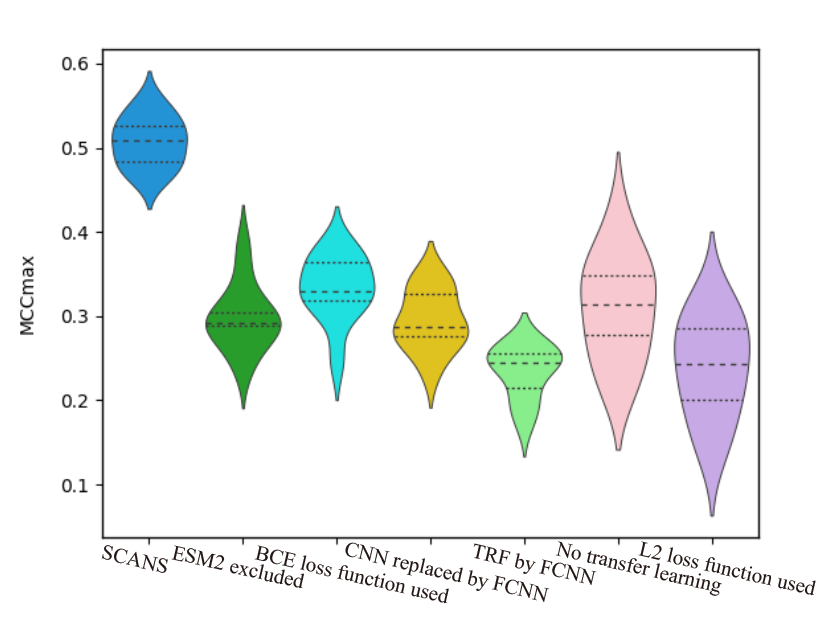  **E** | 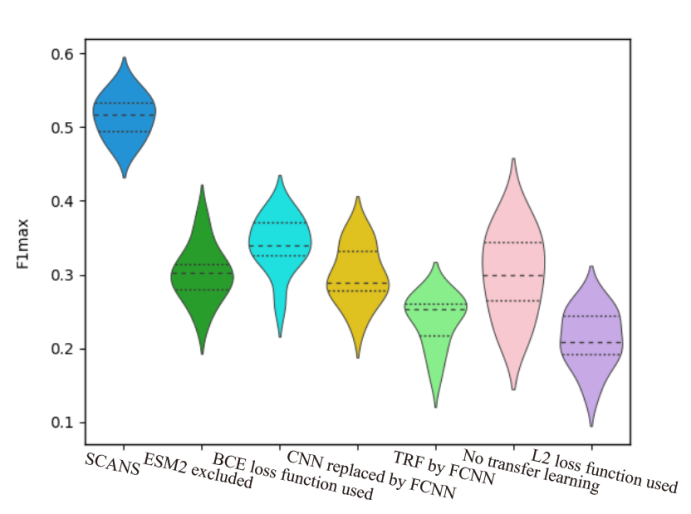  **F** |

**Figure S12**. The violin plot of the MCCmax values the P (panel A), R (panel C), and T (panel E) carbonylation testing datasets. The violin plot of the F1max values on the P (panel B), R (panel D), and T (panel F) carbonylation testing datasets.

| 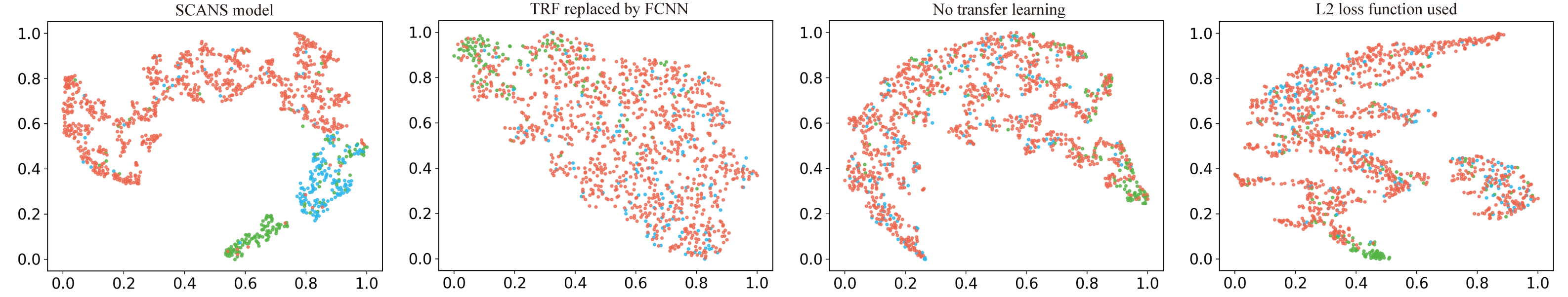  **A** |
| --- |
| 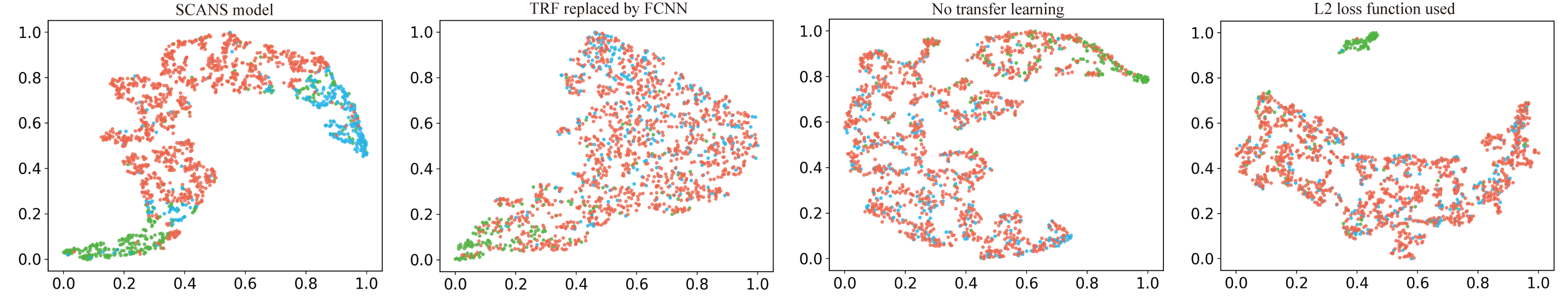  **B** |
| 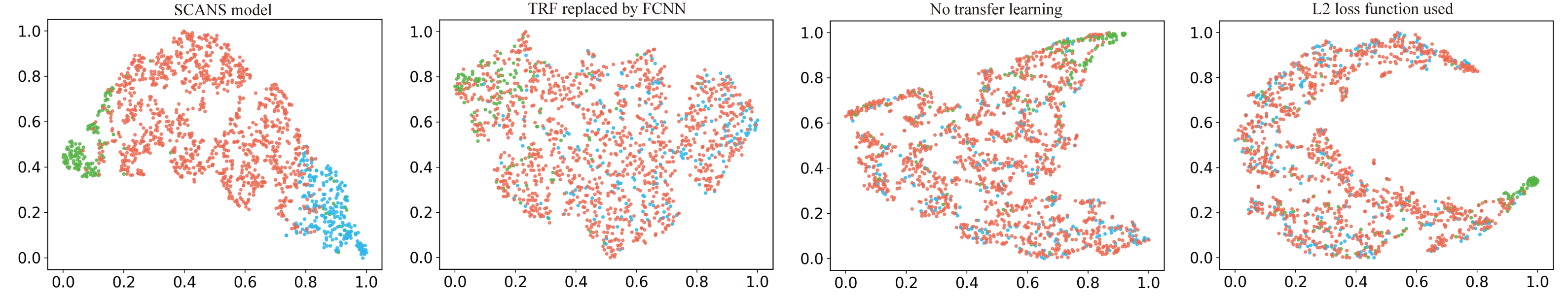  **C** |
| 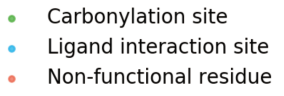 |

**Figure S13**. The t-SNE clustering results of the output features of SCANS and three ablation setups on the P (panel A), R (panel B), and T (panel C) carbonylation testing datasets.

## Experimental settings

SCANS was implemented in python3.7, the core code was built on the PyTorch 1.12.0 framework. SCANS is trained using the Adam optimizer, with the learning rate and batch size set to 0.001 and 256, respectively. All the training, validation and testing procedures were performed based on Nvidia RTX 3070 GPUs with 32GB of memory. The training epoch is set to 100. The best models are in the 47th, 45th, 44th, and 41th epoch for K, P, R, T carbonylation datasets, respectively.

## Data and code Availability

The authors declare that the data supporting the findings of this study are available within the article and its supplementary information files. The source code, benchmark datasets, final models and results are all available at the GitHub (https://github.com/jianzhang-xynu/SCANS). We detail the experimental settings and tutorial of the predictor in the GitHub repository.
